# SpG and SpRY variants expand the CRISPR toolbox for genome editing in zebrafish

**DOI:** 10.1038/s41467-022-31034-8

**Published:** 2022-06-14

**Authors:** Fang Liang, Yu Zhang, Lin Li, Yexin Yang, Ji-Feng Fei, Yanmei Liu, Wei Qin

**Affiliations:** 1 Key Laboratory of Brain, Cognition and Education Sciences, Ministry of Education; Institute for Brain Research and Rehabilitation, South China Normal University, 510631 Guangzhou, Guangdong China; 2 Institute of Modern Aquaculture Science and Engineering, Guangdong Provincial Engineering Technology Research Center for Environmentally-Friendly Aquaculture, Guangdong Provincial Key Laboratory for Healthy and Safe Aquaculture, School of Life Sciences, South China Normal University, 510631 Guangzhou, Guangdong China; 3 Department of Pathology, Guangdong Provincial People’s Hospital, Guangdong Academy of Medical Sciences, 510080 Guangzhou, Guangdong China

**Keywords:** Disease model, CRISPR-Cas9 genome editing

## Abstract

Precise genetic modifications in model organisms are essential for biomedical research. The recent development of PAM-less base editors makes it possible to assess the functional impact and pathogenicity of nucleotide mutations in animals. Here we first optimize SpG and SpRY systems in zebrafish by purifying protein combined with synthetically modified gRNA. SpG shows high editing efficiency at NGN PAM sites, whereas SpRY efficiently edit PAM-less sites in the zebrafish genome. Then, we generate the SpRY-mediated cytosine base editor SpRY-CBE4max and SpRY-mediated adenine base editor zSpRY-ABE8e. Both target relaxed PAM with up to 96% editing efficiency and high product purity. With these tools, some previously inaccessible disease-relevant genetic variants are generated in zebrafish, supporting the utility of high-resolution targeting across genome-editing applications. Our study significantly improves CRISPR-Cas targeting in the genomic landscape of zebrafish, promoting the application of this model organism in revealing gene function, physiological mechanisms, and disease pathogenesis.

## Abstract (web summary)

The recent development of PAM-less base editors makes it possible to assess the functional impact and pathogenicity of nucleotide mutations in animals. Here the authors show that SpG and SpRY could edit NGN and NNN PAMs in zebrafish using the purified protein with synthetically modified gRNA, respectively.

## Introduction

Clinical studies have shown that many genetic diseases are caused by single-nucleotide variants (SNVs), which affect single amino acids instead of causing whole gene disruption^1^. As genomic studies continue to reveal SNVs, functional validation to assess their phenotypic impact in vivo remains a problem. Usually, a cell-based or animal disease model can be used to determine the functional relevance and pathogenic characteristics of SNVs^2^. Because of the complexity of disease phenotype, animal models including zebrafish have unique advantages.

The clustered regularly interspaced short palindromic repeats (CRISPR)/Cas9 system has been successfully harnessed for various genome editing and regulation applications^3^; however, there is still a lack of an efficient method to introduce precise mutations in animal models. The main reason is that the canonical SpCas9 that recognized the 5′-NGG-3′ PAM sequence may not be available near the target of interest, which is a crucial factor for homology direct repair (HDR) and base editing.

To expand the targeting coverage, besides identifying additional naturally occurring CRISPR nucleases that may have different PAM requirements^4,5^, researchers have engineered SpCas9 to recognize other PAM sequences through directed evolution and structure-guided design^6–8^. Until now, many SpCas9 variants such as VQR-Cas9, xCas9, Cas9-NG, and SpCas9 orthologues have been demonstrated to edit the human cell and plant genomes^6,7,9,10^, but fewer SpCas9 variants or orthologues were reported to have genome-editing activities in animal organisms, especially zebrafish. Many CRISPR-based technologies that work at a cellular level cannot be simply transplanted into animals. For example, the CRISPR/Cas12a system can be applied in zebrafish only in the form of a ribonucleoprotein (RNP) complex instead of mRNA^11^; xCas9 and Cas9-NG only exhibited limited editing ability in the zebrafish genome, even in the form of an RNP complex (Supplementary Fig. 1). This has limited the application of zebrafish in the modelling of human genetic diseases, which is of great value in the study of disease pathogenesis and drug screening. Recently, two SpCas9 variants (SpG and SpRY) and their related base editors with more relaxed PAM requirements have been reported to exhibit robust activities with minimal side effects in human cells and plants^8,12–14^. SpG has high genome-editing activity at NGN PAM (where N is A, C, G, or T) sites, whereas SpRY can target almost all PAMs in the genome (with NRN being preferable to NYN, where R is A or G; Y is C or T). However, whether SpG and SpRY nuclease and base editor variants can be used to improve genome editing in zebrafish remains unclear.

Here, we optimized SpG and SpRY by combining the purified protein with synthetically modified gRNA and examined their efficiency at various PAM sites in zebrafish. Our study showed that SpG displayed vigorous activities at NGN PAM sites, but SpRY could edit almost all kinds of PAM sequences in zebrafish. Furthermore, we developed SpRY-based base editors, which readily generated the related base conversions at the target sites with non-canonical PAMs in zebrafish. Collectively, the toolbox developed in this study will significantly increase the density of editable sites in zebrafish and promote the generation of human genetic disease models in zebrafish for pathogenesis and treatment research.

## Results

### Optimized CRISPR-SpGCas9 system exhibits high activities in zebrafish

**Fig. 1:**
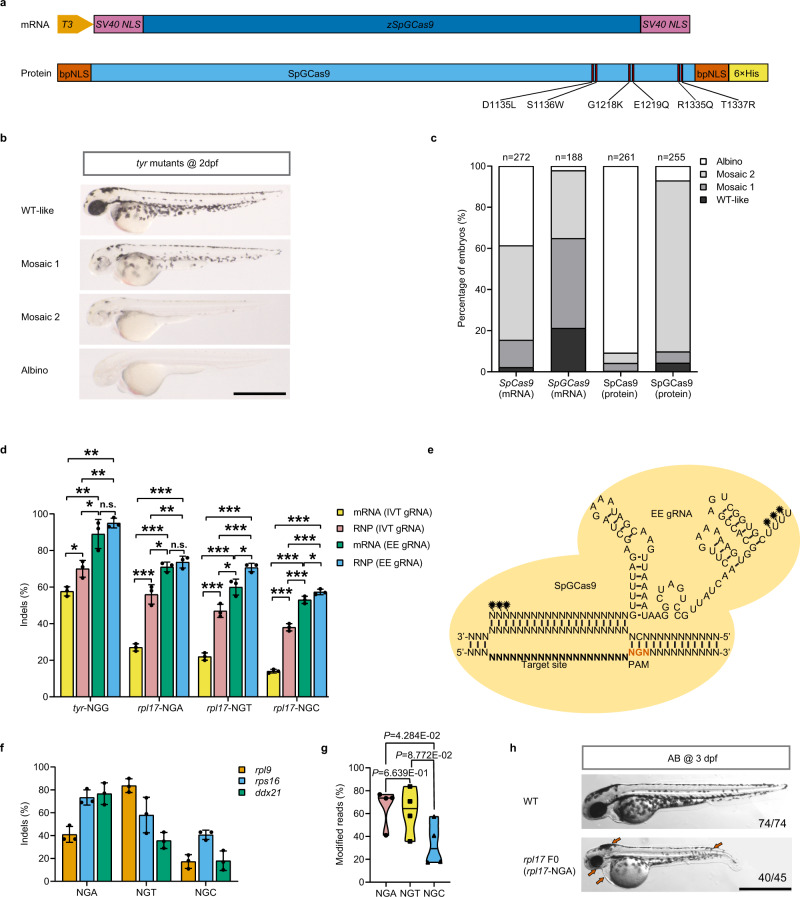
Optimized SpGCas9 showed high editing efficiency at NGN PAM sites in zebrafish. **a** Schematic illustration of codon-optimized SpGCas9 mRNA (zSpGCas9) and prokaryotic-expressed SpGCas9 protein. Six specific amino acid mutations (D1135L, S1136W, G1218K, E1219Q, R1335Q, and T1337R) were introduced into the SpCas9 amino acid sequence to obtain SpGCas9. **b** Biallelic disruption of tyrosinase (*tyr*) by SpGCas9 generates mosaic pigmentation phenotypes. Lateral views of 2 dpf embryos are shown. The mosaic pigmentation degree (WT-like, mosaic 1, mosaic 2, albino) compared with wild-type (WT) is defined in Supplementary Fig.3a. Scale bars: 1 mm. This experiment was repeated 3 times independently with similar results. **c** Phenotype statistics of embryos injected with SpCas9 mRNA/gRNA duplex, SpGCas9 mRNA/gRNA duplex, SpCas9: gRNA RNP complex, and SpGCas9: gRNA RNP complex. The stacked columns indicate the percentages of albino (white), mosaic 2 (light grey), mosaic 1 (medium grey), and WT-like (dark grey) zebrafish related to **b**. The total number of embryos was shown above each column. **d** Bar plot of indel efficiency edited by SpGCas9 mRNA/gRNA duplex or SpGCas9: gRNA RNP complex containing IVT gRNAs (in vitro-transcribed gRNA) or EE gRNAs (EasyEdit gRNA) across *tyr*-NGG, *rpl17*-NGA, *rpl17*-NGT and *rpl17*-NGC PAM sites. Editing efficiency was assessed by targeted Sanger sequencing and ICE analysis (values are presented as mean value ± standard deviation (SD), *n* = 3 biological replicates). Data were analysed by two-tailed paired *t* test. *P ≤ 0.05, **P ≤ 0.01, and ***P ≤ 0.001 (n.s. not significant). The exact *P* values are listed in Supplementary Data 6. **e** A schematic diagram of the sequence and secondary structure of EE gRNA loaded into SpGCas9 protein and bound to the genomic target site. The chemically modified nucleotides are labelled with black stars. **f** The editing efficiency of SpGCas9 at 9 targets with NGH PAMs in the genes *rpl9*, *rps16* and *ddx21* (values are presented as mean value ± standard deviation (SD), n = 3 biological replicates). **g** Assessment of the preference of SpGCas9-mediated mutagenesis for the last N of NGN PAMs using the violin plot. Each data point represents the averaged editing activity at the particular site. The centre line shows means of all data points. Two-tailed paired *t* test were performed (with *P* values marked). **h** Lateral views of SpGCas9:*rpl17* EE gRNA (targeting NGA PAM) RNP complex induced mutated F0 at 3 dpf. Red arrows indicate the specific features of *rpl17* mutants. Scale bar: 1 mm. this experiment was repeated 3 times independently with similar results. All source data in this figure are provided as a Source data file.

To test the activity of SpGCas9 in zebrafish, we first constructed the zebrafish codon-optimized SpG variant (Fig. 1a). To directly observe the activity of SpGCas9, the tyrosinase (*tyr*) gene with a previously published gRNA targeting a 5′-NGG-3′ PAM was chosen^15^. A loss-of-function mutation in *tyr* results in the loss of eye and body pigment in zebrafish^16^. After we injected the same amount of SpGCas9 mRNA into the zebrafish one-cell-stage embryos, SpGCas9 mRNA worked, but with a lower proportion of embryos displaying an albino-like phenotype than SpCas9 mRNA (Fig. 1b, c). A previous study demonstrated that using SpCas9 RNP (Cas9: gRNA ribonucleoprotein) complex instead of mRNA could increase the indel frequency in zebrafish^17^. Therefore, we purified the SpG protein with bpNLS at both terminals instead of SV40 NLS (Fig. 1a and Supplementary Fig. 2). The effective concentration of the RNP complex is an essential factor for Cas9 system efficiency in vivo, especially for model organisms that need an injection, such as zebrafish. We observed a noticeable enhancement in activity along with increased concentrations of SpGCas9 RNP complex, as evidenced by zebrafish phenotype analysis (Supplementary Fig. 3). We chose 5 μM as the final usage in the following experiments. Not surprisingly, injecting the SpCas9 or SpGCas9 RNP complex into embryos indeed improved the cleavage activity in zebrafish (Fig. 1c).

Then, we chose three other sites with NGA, NGT and NGC PAM in *rpl17* locus to check whether the SpG protein can improve the activity. Although the SpG protein can enhance the indel frequency, we observed that cleavage activity was a little low for the *rpl17* locus (Fig. 1d). To further improve the editing efficiency of the SpGCas9 system, we modified the gRNA terminals with 2′-O-methyl-3′-phosphorothioate (MS) to increase its stability, as previously described^18^ (Fig. 1e). Our results showed that MS-modified gRNAs (EasyEdit sgRNA, EEgRNA) dramatically increased the targeting efficiencies of SpGCas9 at four different PAM sites compared with unmodified gRNAs transcribed in vitro (IVT gRNA), whether using mRNA or RNP complex (Fig. 1d). Among various combinations, protein plus MS-modified gRNA performed best. Therefore, in the following experiments, the purified Cas9 protein combined with MS-modified gRNA was used unless otherwise stated.

To further investigate the editing activity of SpGCas9 in zebrafish, we selected 9 targets with NGH (where H is A, C or T) PAM in three genes, including *rpl9*, *rps16* and *ddx21*. After injection of SpGCas9 RNP complex, three groups of larvae, each with six embryos, at 48 h post-fertilization were randomly selected and mixed for genomic DNA extraction. PCR amplification of the region covering the target site was performed, and the products were directly used for Sanger sequencing. Then, the indel mutation frequency was analysed using Synthego’s Inference of CRISPR Editing (ICE) tool^19^. The results showed that SpG could edit all NGH PAM sites tested, albeit with variable efficiencies (41.0–76.7% at the three NGA PAM sites, 35.7–83.7% at the three NGT PAM sites, and 17.3–40.67% at the three NGC PAM sites, Fig. 1f, g). Our previous study demonstrated that loss of ribosomal protein (RP) genes in zebrafish often results in a small head, small eyes, and enlarged hindbrain ventricles^20,21^. The same phenotype was observed in *rpl17*-NGA PAM knockout F0 embryos (Fig. 1h) and in most other RP gene targeting groups (Supplementary Data 1). Moreover, by analysing the Sanger sequencing chromatograms of the target regions (*rpl17*-NGA site) using ICE (Supplementary Fig. 4a), we found that the DNA damage pattern induced by SpG was similar to that induced by SpCas9, with short insertions or deletions^22^ (Supplementary Fig. 4b). For off-target analysis, we chose three sites to evaluate the effect. For every single gRNA, the three most likely off-target sites were predicted by Cas-OFFinder^23^ and CRISPOR^24^, then the results were analysed by CRISPResso2^25^ through next-generation sequencing (NGS). Only low off-target mutations (≤0.5%) were found in the *rpl17*-NGA group but no off-target mutations for the other 2 gRNAs (Supplementary Table 1). Taken together, these results indicate that, after optimization, SpG is an efficient and broadly targeting nuclease across NGN PAM sites in zebrafish.

### Optimized SpRY nuclease could target almost all kinds of PAM sequences in zebrafish

**Fig. 2:**
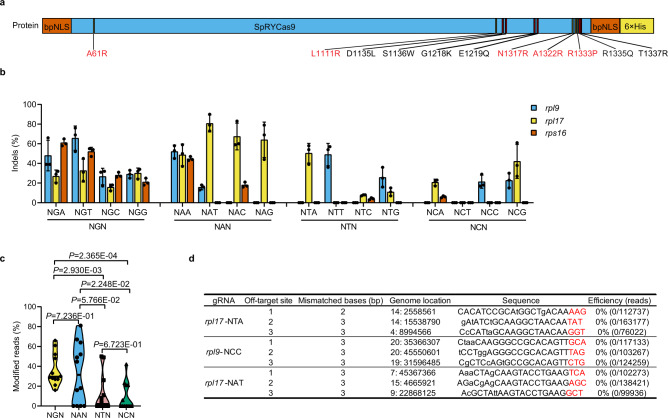
Optimized SpRYCas9 nuclease targeted different NNN PAMs in zebrafish. **a** Schematic illustration of the amino acid mutations that developed SpRYCas9 from SpGCas9. Five more amino acid mutations (A61R, L1111R, N1317R, A1322R, and R1333P; in red) were introduced into SpGCas9 to obtain SpRYCas9. **b** The editing efficiency of 48 gRNAs targeting NNN PAMs of *rpl9*, *rpl17*, and *rps16* in zebrafish. (Values are presented as mean value ± standard deviation (SD), *n* = 3 biological replicates). **c** Assessment of the preference of SpRYCas9-mediated mutagenesis for the second N of NNN PAMs using the violin plot based on the data in **b**, Each data point represents the averaged editing activity at the particular site. The centre line shows means of all data points. Two-tailed paired *t* test were performed (with *P* values marked). **d** Detection of mutation at potential off-target sites induced by SpRYCas9 nuclease at three loci using NGS. The PAM sequences are underlined in red. All source data in this figure are provided as a Source data file.

In human cells, SpRY was reported to have efficient genome editing activity at NRN PAMs and exhibited tolerance to NYN PAMs at various loci^8^. To test the nearly unconstrained PAM requirement of SpRY in zebrafish, we introduced five amino acid mutations (A61R/L1111R/N1317R/A1322R/R1333P) into SpG to generate SpRY (Fig. 2a and Supplementary Fig. 5). Following the same experimental procedure mentioned above, the nuclease activity and PAM preference of SpRY were thoroughly evaluated in zebrafish. Forty-eight endogenous genomic sites bearing all 16 possible alternative PAM sequences from three genes (*rpl9*, *rpl17* and *rps16*) were tested. As indicated in Fig. 2b, SpRY exhibited efficient editing at all 12 NGN (15.67–65.67%) and 8 of the 12 NAN PAM (15.50–80.67%) sites, 4.0–50.3% editing efficiency at 6 of 12 NTN PAM sites, and a frequency between 6.0 and 42.0% at 5 of the 12 NCN PAM sites. The average editing activities of SpRY against NGN and NAN PAM were 34.3 and 41.0%, respectively. In contrast, the average editing activities for NCN and NTN PAM were 18.0 and 13.3%, respectively (Fig. 2c). SpRY showed a higher preference for NRN than NYN in zebrafish, which is consistent with the results reported in human cells and plants^8,12^. Considering PAM-flexible Cas9 variants may increase the risk of off-target editing, just like we did in SpGCas9 nuclease, we analysed the off-target effects of three gRNAs with relatively high cleavage activities. No off-target mutations were found in all the groups (Fig. 2d).

Previous studies have shown that efficient germline transmission occurs in SpCas9-induced mutations in zebrafish^16^. To determine whether the germline transmission of SpG- and SpRY-induced mutations was equally efficient, we randomly intercrossed three pairs of *tyr*-NGT-site founders to generate F1. All six founders were confirmed to be heritable through the observation of albino F1 embryos. At another three sites (*ddx21*-NGC, *ddx21*-NGT, and *rpl17*-NGA), the founders (11/11, 3/3, and 8/10, respectively) could generate heritable F1 embryos when outcrossing them with wild-type fish. Moreover, we randomly selected one positive F0 founder from each site and analysed the germline transmission rate. A germline transmission rate of up to 62.5% was achieved (Supplementary Table 2). Therefore, germline transmission of the mutations induced by SpG and SpRY was as efficient as those induced by SpCas9^16^.

### SpRY-CBE4max mediates cytosine base editing at PAM-less sites in the zebrafish genome

**Fig. 3:**
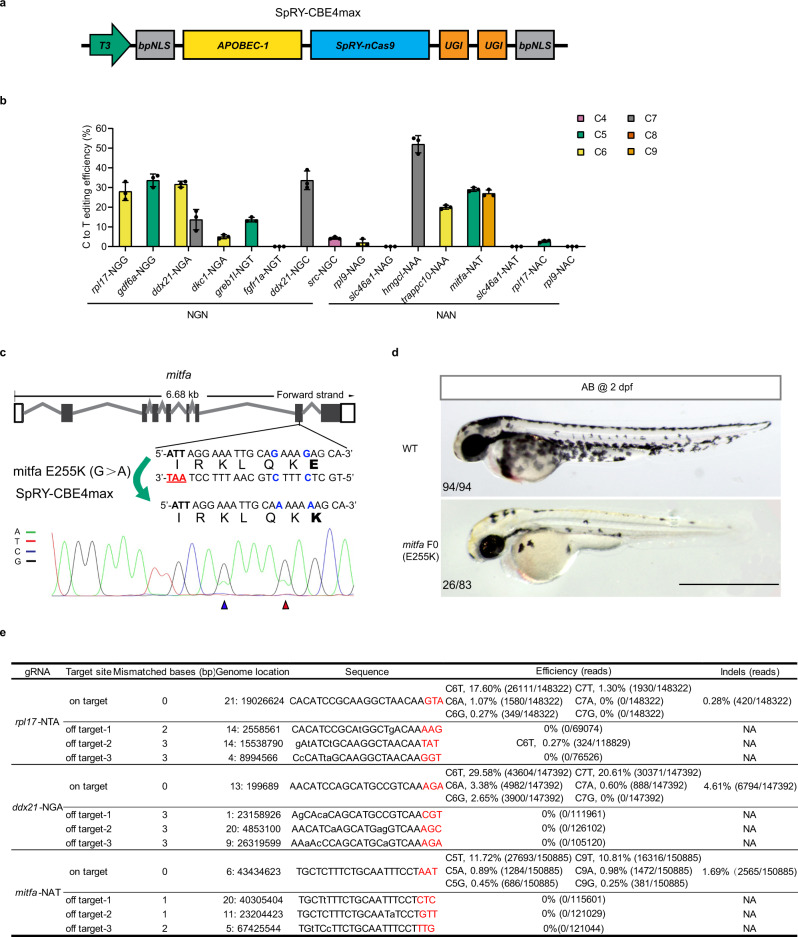
Efficient cytosine base editing mediated by SpRY-CBE4max across various PAMs. **a** The mRNA construct of SpRY-CBE4max used for cytosine base editing in zebrafish. **b** Summary of C-to-T base editing efficiency of various loci with NRN PAMs induced by SpRY-CBE4max editor in zebrafish. The position of the editing base in the gRNA was labelled with numbers. (Values are presented as mean value ± standard deviation (SD), *n* = 3 biological replicates) **c** Schematic diagram and sequencing results of *mitfa* (E318K) mutation induced by SpRY-CBE4max. The PAM sequences are underlined in red, the detected nucleotide changes are highlighted in blue, the targeted amino acids are highlighted in bold, the nucleotide substitutions are indicated by a red arrowhead in the sequencing chromatograms, and the synonymous mutations induced by SpRY-CBE4max are indicated with a blue arrowhead. **d** Lateral view of 2 dpf F0 embryos injected with *mitfa* (E318K) gRNA and SpRY-CBE4max mRNA showing pigmentation defects. Scale bar: 1 mm. This experiment was repeated three times independently with similar results. **e** On-target, product purity and off-target analysis of SpRY-CBE4max induced C-to-T editing at *rpl17*-NTA, *ddx21*-NGA and *mitfa*-NAT sites using NGS. The PAM sequences are underlined in red. NA: Not Applicable. All source data in this figure are provided as a Source data file.

Some studies, including ours, reported that cytosine base editors (CBEs) can be efficiently applied in zebrafish^26,27^. However, the PAM preference plus narrowed targeting window essentially limited their targeting scope in zebrafish. To date, several CBEs with different PAM preferences were reported to be active in cultured cells, of which SpRY-CBE4max was the most recently identified flexible variant^8^. Considering that the PAM-less feature of SpRY allows for base editing of many previously inaccessible bases, we tested its compatibility with CBE in zebrafish. After SpRY-CBE4max mRNA (Fig. 3a) and related gRNA were injected into zebrafish embryos, the base-editing activity of SpRY-CBE4max was then investigated in zebrafish in 16 genes with 32 sgRNAs that target non-canonical PAMs. Our results showed that the C-to-T base substitutions could be detected at 17 of 32 sites, although the editing efficiencies varied (Fig. 3b and Supplementary Fig. 6). These data indicated that SpRY could greatly expand the targeting scope of CBE in zebrafish.

Melanocyte-inducing transcription factor (MITF) is the master melanocyte transcription factor and has a complex role in melanoma^28^. Loss of *mitfa* leads to almost complete loss of melanocytes in zebrafish^29^. A Glu318Lys variant of MITF was reported in individual patients with cutaneous melanoma^30,31^. To construct a genetic variant-related disease model, one gRNA site with a NAT PAM was selected. After injecting the SpRY-CBE4max mRNA and *mitfa* gRNA into zebrafish embryos, we assessed the base conversion. The overlapping C/T peaks at the targeted C (position 5 from the 5′ end) could be detected in pools of 10 randomly selected embryos. Besides the expected C, the bystander C (position 9 from the 5′ end) could also be edited (Fig. 3c). Because this bystander editing causes synonymous mutation, this accidental editing will not further change the amino acid sequence of *mitfa*. Moreover, the injected embryos showed the phenotype of pigment deficiency at 2 dpf (days post-fertilization), which reflects the importance of E255K to keep *mitfa* function (Fig. 3d).

To investigate the product purity, indels and off-targets of SpRY-CBE4max in zebrafish, three loci were chosen and analysed through NGS. The results showed that besides the mainly C to T conversion, undesired by-products (C-to-A and C-to-G conversion) were detected at a low efficiency (<4%) (Fig. 3e and Supplementary Figs. 7–9), which is consistent with previous reports^32,33^. In addition, the indels could also be observed at all three sites (0.28–4.61%) and only in the *rpl17*-NTA group low off-target editing (0.27%) was detected (Fig. 3e). What is more, high germline targeting efficiency and germline transmission rate were observed (Supplementary Table 3). These results demonstrate the powerful application of SpRY-CBE4max as a PAM-less base editor for disease modelling in zebrafish.

### zSpRY-ABE8e enables efficient adenine base editing in a wide range of the zebrafish genome

**Fig. 4:**
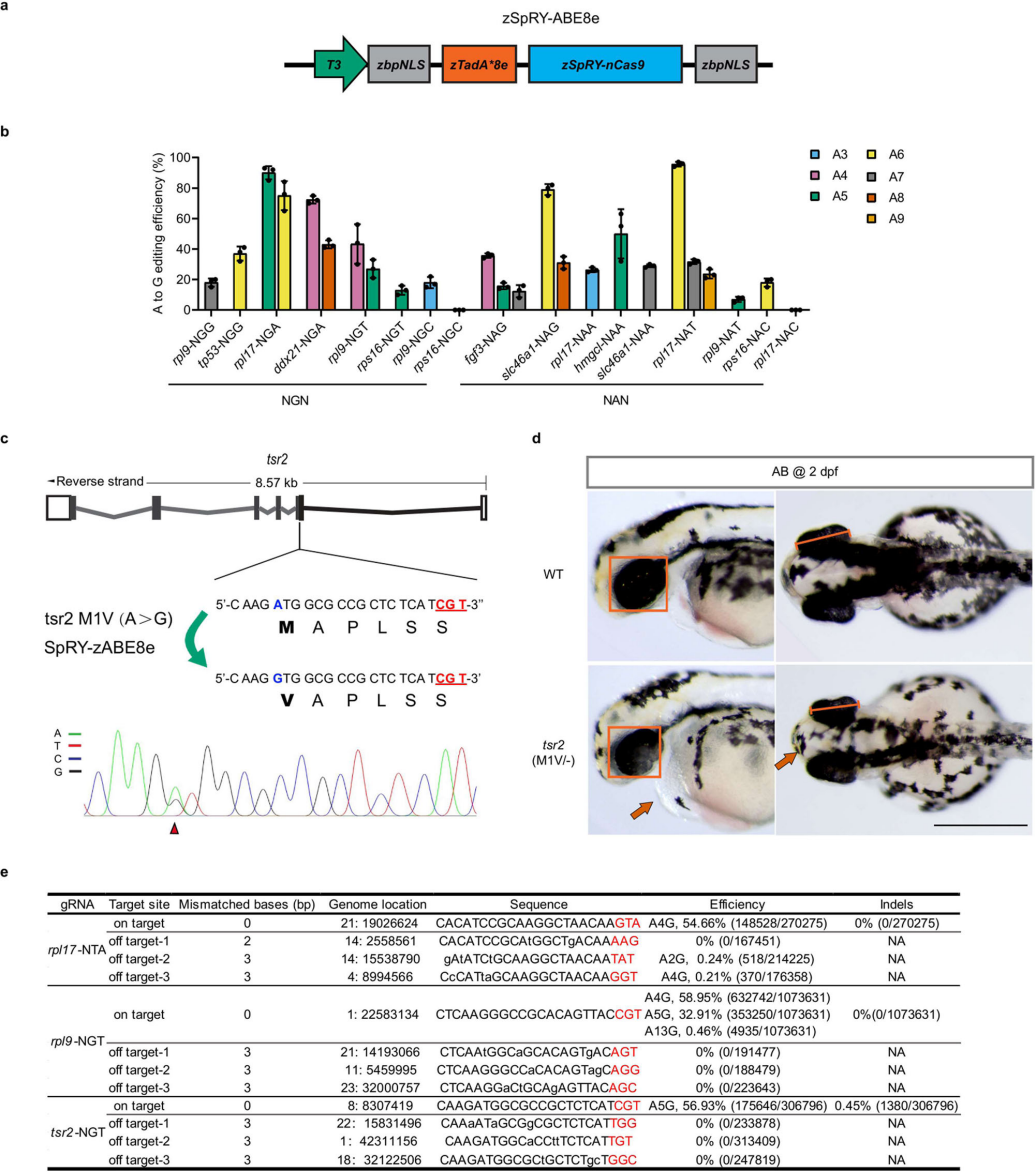
Efficient adenine base editing mediated by zSpRY-ABE8e across various PAMs. **a** The mRNA construct of zSpRY-ABE8e used for adenine base editing in zebrafish. **b** Summary of A-to-G base editing efficiency of various loci with NRN PAMs induced by zSpRY-ABE8e editor in zebrafish. The position of editing base in the gRNA was labelled with numbers. (Values are presented as mean value ± standard deviation (SD), *n* = 3 biological replicates.) **c** Schematic diagram and sequencing results of *tsr2* (M1V) mutation induced by zSpRY-ABE8e in zebrafish. The PAM sequences are underlined in red, the detected nucleotide changes are highlighted in blue, the targeted amino acids are highlighted in bold, and the nucleotide substitutions are indicated with a red arrowhead in the sequencing chromatograms. **d** Morphological phenotype of *tsr2* ^(*M1V*)/^^−^-deficient embryos at 2 dpf. The comparison of eye size is underlined with a red frame in the lateral view and with diameter measurement in the dorsal view. Red arrows indicate a pointy head and slight pericardial oedema of *tsr2* ^(*M1V*)/−^ mutants. Scale bar: 500 μm. This experiment was repeated three times independently with similar results. **e** On-target, product purity and off-target analysis of zSpRY-ABE8e induced A-to-G editing at *rpl17*-NTA, *rpl9*-NGT and *tsr2*-NGT sites using NGS. The PAM sequences are underlined in red. NA: Not Applicable. All source data in this figure are provided as a Source data file.

Adenine base editors (ABEs), such as zABE7.10 and zABEmax, have been reported to perform A-to-G base editing in zebrafish, but the narrowed targeting window and locus-dependent manner severely limited their application^34^. Recently, an evolved ABE8e was reported to have robust A-to-G editing efficiency in human cells; it catalyses deamination >1000 times faster than early ABEs^35^. Therefore, we investigated whether SpRY-ABE8e could improve adenine base editing in zebrafish. After synthesizing TadA8e, the core component of ABE8e optimized by the zebrafish codon, was fused to the 5-terminal of SpRY (D10A) nickase to generate a new construct, zSpRY-ABE8e (Fig. 4a).

The endogenous nine genes (*rpl9*, *rpl17*, *ddx21*, *fgf3*, *tp53*, *slc46a1*, *shroom4*, *hmgcl* and *rps16*) were used to test zSpRY-ABE8e’s activity toward various PAMs in zebrafish. Strikingly, zSpRY-ABE8e showed detectable A-to-G editing across most of the relaxed PAM sites in zebrafish, with the highest frequency up to 96% (Fig. 4b and Supplementary Fig. 10). The highly active editing window for zSpRY-ABE8e appeared to span from the third to the ninth nucleotide along the protospacer (Supplementary Fig. 11), which was consistent with ABE8e editing data in human cells^35^. These results indicate that zSpRY-ABE8e can efficiently induce A-to-G conversions in a wide editable range of the zebrafish genome, including PAM-less sites.

A new strategy for gene disruption, i-Silence, was recently achieved by introducing a mutation to the start codon (ATG to GTG or ACG) using the ABE system^36^. Taking advantage of the i-Silence strategy, with zSpRY-ABE8e, we successfully disrupted zebrafish *tsr2*, a Diamond-Blackfan anaemia (DBA) disease-causing gene^37^, creating a new DBA model in zebrafish. Sanger sequencing results showed that overlapping A/G peaks at the targeted A of the start codon could be detected (Fig. 4c). When F0 founders mated to a previously generated *tsr2* heterozygous mutant adult, several embryos exhibited small eyes, pointy head and slight pericardial oedema at 2 dpf (Fig. 4d), and further genotyping confirmed these embryos contained the M1V mutation. Compared with CBE system, through NGS analysis, the zSpRY-ABE8e system has a high product purity and the indels at all three sites could barely be detected (Fig. 4e and Supplementary Figs. 12–14), which is quite different from the zABE7.10 system^34^. Moreover, this system also exhibited a high germline targeting efficiency and germline transmission rate (Supplementary Table 3). These data demonstrated that zSpRY-ABE8e can be used as an efficient and precise gene disruption tool for functional genetic studies and disease model generation in zebrafish.

## Discussion

PAM preference is the critical limiting factor for every CRISPR-based targeted genome editing tool, especially for HDR, because it requires that the site cut by nuclease be as close as possible to the desired location at a given site^38^. Therefore, reducing or eliminating the PAM requirement of Cas nucleases will dramatically advance various CRISPR technologies. Here, we optimized SpG and SpRY, and characterized their targeted genome editing in zebrafish. Our results indicate that both SpG and SpRY can edit the zebrafish genome with high targeting efficiency. We adopted two strategies (purified bpNLS-containing proteins and MS-modified gRNA) to enhance their nuclease activity in zebrafish. It has been reported that using the SpCas9 protein instead of mRNA can more efficiently target and edit DNA in zebrafish embryos^39^. We observed the same phenomenon for SpRY nuclease in zebrafish embryos (Supplementary Fig. 15). The MS-modified gRNA exceeded our expectations by dramatically improving the editing frequency at several loci (Supplementary Fig. 16). Collectively, using our optimized approaches, purified bpNLS-containing proteins and MS-modified gRNA, SpRY can display efficient genome editing in a nearly PAM-less manner in zebrafish.

Although SpRY can target any NNN PAM in human cells, PAM preference does exist^8^. In zebrafish, we observed the same PAM preference (NRN is preferable to NYN). However, toward a specific locus with NYN PAM (*rpl9*-NTT or *rpl17*-NCG), SpRY-mediated editing efficiency was not low (both above 40%), which means it is possible to find a proper site suitable for NYN PAM with some trials. Our data revealed that, except for NCT PAM, all other NYN PAM sites could be targeted at least at one of two loci. This does not mean that NCT PAM cannot be accessed in zebrafish because our data are limited. Besides the gRNA sequence itself, many other factors such as gRNA secondary structure and chromatin accessibility have been reported to influence the CRISPR/Cas9 efficiency in zebrafish^40,41^. Therefore, we think whether SpRY can edit a site is a complex locus-dependent issue.

As SpRY can target almost all PAMs in zebrafish, its off-target effects may be high during genome editing. However, we did not observe its obvious off-target effects in zebrafish (Figs. 2d, 3e, and 4e), probably due to our limited data. Even if we encounter serious off-target effects at some sites, we can still use the following aspects to solve this problem. First, Cas9 RNP delivery instead of mRNA or plasmid has been proved to alleviate potential off-target editing in human cells^42^. What we optimized in this study is exactly the protein itself. Second, a rigorous design of the gRNA is another way to avoid off-target cleavage, which could be achieved using algorithms and websites that predict potential off-targets^23,43^. Third, the high-fidelity variant of SpRY with improved genome-wide specificity has been reported to reduce off-target editing in human cells;^8^ therefore, the same strategy can be applied to zebrafish. In addition, several CBE and ABE variants with reduced off-target RNA editing activity have been developed using the structure-guided principles^44–46^, which provide an effective method to solve this problem in zebrafish. Last, in zebrafish, this potential off-target problem can be solved by consistently selecting targeted mutant embryos with specific phenotypes through multiple generations of breeding.

For precise disease modelling, the application of CRISPR/Cas9 to generate point mutations into zebrafish can be of particular value. Besides base editors, homology-directed repair is the most commonly used method to generate point mutations in zebrafish. Although the HDR-based modification has been widely used in human cells and mammals, the efficiency is still very poor in zebrafish and it has a strong locus-dependent problem^47^. Generally speaking, the efficiencies of HDR is far less than base editors. Our experiment also proved that the editing efficiency (0.68% ± 0.07% vs 11.72% ± 0.53%) is significantly lower than SpRY-CBE4max induced base conversion at the same locus (Supplementary Fig. 17). Considering the convenience and high efficiencies of base editors, the first choice will be base editors for these four types of transition mutations (C to T, G to A, A to G and T to C). Although HDR-based methods have been optimized by many groups in zebrafish^48–50^, the efficiency is still limited, probably due to the complexity of HDR. A previous study showed an inverse relationship between HDR efficiency and the distance between the insertion site and Cas9: gRNA RNP cut site^38^. The PAM-less SpRY variant revealed the possibility that the cut site is very close to the insertion site at a given locus, which could theoretically significantly facilitate precise editing in vivo.

Although base editors reported in this study can target relaxed various PAMs, there are still some limitations, especially for CBE. Surprisingly, SpG-CBE4max did not show any activities at the sites (PAM = NGN) that could be targeted by SpRY-CBE4max, the phenomenon was also observed by another group^51^. In contrast, the zSpG-ABE8e has the activities to induce A to G conversions, the efficiencies were comparable to zSpRY-ABE8e at the same loci (Supplementary Fig. 18). What’s more, the cytidine deaminase that SpRY-CBE4max contained is rat APOBEC1. As APOBEC1 preferentially targets the cytidines preceded by T (namely the cytidines in the TC motif), the editing scope of CBE is further limited. In addition, the product purity and indels are still problems of CBE. Recently, some evolved CBEs have been reported to have expanded target compatibility and improved activities^52^, so the new tools may solve the problem encountered by SpRY-CBE4max. Another limitation is that the base editors reported here can only efficiently induce the four transition mutations (C to T, G to A, A to G and T to C), but cannot perform the other eight transition mutations. Recently, prime editing using ribonucleoprotein complexes has been reported to have activities in zebrafish^53^. Although the efficiency is still low, it has strong complementary strengths for base editing towards other eight transition mutations. It means if the intended base conversion cannot be accessed by base editors, the prime editing or HDR will be your choice.

By combining the purified bpNLS-containing proteins and MS-modified gRNA, we revealed that nearly all PAM-less sites could be efficiently targeted by SpRY in the zebrafish genome. Furthermore, SpRY-CBE4max and zSpRY-ABE8e could efficiently target previously inaccessible sites in zebrafish. The gene-editing toolbox developed here will expand zebrafish applications to reveal gene function, physiological mechanisms, and disease pathogenesis and treatment.

## Methods

### Zebrafish maintenance

Wild-type AB line embryos were raised at 28.5 °C and staged according to the description^54^. The selection of mating pairs (12–15 months) was random from a pool of 30 males and 30 females. All animal protocols were reviewed and approved by the University Animal Care and Use Committee of the South China Normal University.

### Plasmid construction and mRNA generation

pT3TS-zSpGCas9 plasmid, including D1135L/S1136W/G1218K/E1219Q/R1335Q/T1337R modifications, was constructed based on plasmid pT3TS-zSpCas9 (Addgene, #46757). Plasmid pT3TS-zSpRYCas9 was further constructed with additional five mutations (A61R/L1111R/N1317R/A1322R/R1333P). For plasmid SpRY-CBE4max, the coding sequence of CBE4max-SpRY was cloned from plasmid RTW5133 (Addgene, #275510) and then inserted into vector pT3TS. To generate zSpRY-ABE8e, a fragment containing zebrafish-codon optimized TadA8e and a linker was synthesized by Sangon Biotech and subcloned into plasmid pT3TS-zSpRYCas9 (D10A nickase). All mutations were generated using Vazyme Mut Express II Fast Mutagenesis Kit V2 with primers listed in Supplementary Data 2. All capped mRNAs were in vitro transcribed from an *Xba*I linearized template using T3 mMESSAGE mMACHINE kit (Ambion) and then purified using RNeasy FFPE kit (Qiagen).

### SpGCas9 and SpRYCas9 protein purification

Using pET-28b-Cas9-his plasmid as a scaffold, SpGCas9 and SpRYCas9 coding sequences flanked by bpNLS signals were cloned into pET28b (+) to generate the SpGCas9 and SpRYCas9 protein expression plasmids using ClonExpress II One Step Cloning Kit (Vazyme). All the Cas9 plasmids were transfected into *E. coli* strain BL21 Rosetta 2 (DE3). The cells were then grown in 15 ml LB medium with 50 mg/l kanamycin at 37 °C overnight. Starter cultures were inoculated into a 2 l LB medium containing kanamycin. The cells were grown at 37 °C until the absorbance A600 reached 0.6. Cultures were then rapidly cooled down to 18 °C and induced with 0.2 mM IPTG before shaking at 150 × *g* for another 20 h. Cell pellets were harvested by centrifugation at 4000 × *g* for 10 min and resuspended in 50 ml lysis buffer containing 50 mM NaH_2_PO_4_ pH8.0, 300 mM NaCl, 10 mM imidazole, 1 mM TCEP, 1 mM PMSF and 0.1% Triton X-100. The cell suspension was lysed using a high-pressure cell crusher (Pressure value at 900 bar) for 10 min at 4 °C. The cell lysate was centrifuged for 25 min at 15,000 × *g* at 4 °C and filtered through a 0.45 μm PES membrane. The supernatant was added to 1.5 ml of Ni-NTA Agarose (QIAGEN) that was pre-equilibrated with 5 column volume (CV) lysis buffer. The protein-bound resin was washed with 10 CV wash buffer 1 (50 mM NaH_2_PO_4_ pH8.0, 300 mM NaCl, 1 mM TCEP and 0.1% Triton X-100) and wash buffer 2 (50 mM NaH_2_PO_4_ pH8.0, 300 mM NaCl, 1 mM TCEP and 20 mM imidazole) successively. Cas9 proteins were eluted with a gradually increased concentration of imidazole (30~250 mM) in wash buffer 2 and collected in a tube every 1.5 ml. All eluted fractions were visualized using SDS-PAGE with Instant-Bands (EZBiolab). Fractions were dialysed using Spectra/Pro Float-A-Lyzer G2 (20kD) in SEC buffer (20 mM HEPES pH7.4, 150 mM KCl, 1 mM TCEP, 5% glycerol) and concentrated with a 100 MWCO Amicon® Ultra-2 ml Centrifugal Filters (Millipore). Protein concentrations were measured with BCA assay kit (Pierce^TM^), and proteins were then stored at −80 °C.

### gRNA generation

All the IVT sgRNAs templates in this study were prepared according to the cloning-independent sgRNA generation method^55^ and then transcribed in vitro using T7 MAXIscriptTM Kit (Ambion) and purified with SanPrep Column microRNA Extraction Kit (Sangon Biotech). For EE gRNAs, each gRNA with chemical modifications comprising MS at both ends was synthesized by GenScript and was dissolved as a 1000 ng/μl stock solution and stored at −80 °C. Target sequences are listed in Supplementary Data 3.

### Cas9 RNP complex preparation, microinjection and image acquisition in zebrafish

To prepare the gRNA and Cas9 protein complex, individual gRNA was incubated with Cas9 protein at a molar ratio of 1.2:1 in the reaction buffer (20 mM HEPES, 100 mM NaCl, 5 mM MgCl_2_, 0.1 mM EDTA, pH6.5) at 37 °C for 15 min. One-cell stage zebrafish embryos were injected with 2 nl of a solution containing 5 µM RNP complex or 300 ng/μl Cas9 mRNA and 200 ng/μl gRNA mixture. For SpRY-CBE4max or zSpRY-ABE8e mRNA, 400 ng/μl was used. For the HDR experiment, 300 ng/μl Cas9 mRNA, 200 ng/μl gRNA and 20 ng/μl donor templated were mixed and injected. After 2 or 3 dpf, embryos were anaesthetised with 0.03% Tricaine (Sigma-Aldrich) and mounted in 4% methylcellulose. Images were taken by SZX2-FOF microscope (OLYMPUS) with either an XM10 digital camera (OLYMPUS) or AxioCam MRc5 digital camera (Leica) and edited by Adobe Photoshop CC software.

### Indels mutation analysis

For SpG- or SpRY-induced cleavage experiments, pooled genomic DNA was extracted from 3 pools, each containing 6 randomly collected embryos. The targeted genomic locus was amplified with the primers in Supplementary Data 2. The amplified targeted genomic DNA was purified for Sanger sequencing. Then we uploaded the Sanger sequencing data to the online CRISPR analysis tool-Inference of CRISPR Edits (ICE) (Synthego Co.)^19^ and specified the guide sequence. Once all the samples have been uploaded, click “Add sample to Analysis” to perform the CRISPR editing efficiency analysis.

### Base editing analysis

For all the base editing experiments, 3 pools of 10 randomly selected embryos were collected. The PCR products were directly sequenced and the Sanger sequencing results were analysed by EditR (1.0.10) program^56^.

### HDR template design

A 120 bp single-stranded oligo deoxynucleotide (ssODN) was designed with the following sequences and synthesized by Sangon. Except for the intended A (red), the donor template contained several synonymous mutations (blue). These substitutions avoid possible Cas9 cleavage of the newly edited endogenous DNA sequence. *mitfa* template with mutations:

CACAGAGACATGAGGTGGAATAAAGGCACCATCCTGAAAGCATCAGTGGATTACATTAGGAAgcTtCAGAAAaAGCAGCAGAAAGCAAAAGAGCTGGAAAACAGACAGAAGAGACTAGAA

### Next-generation sequencing (NGS) and analysis

Extracted genomic DNA from wild-type or injected embryos were performed by standard protocol. NGS library was constructed using genomic DNA as a template through two rounds of PCR. First-step PCR amplification of 100–270 bp sequences from on/off-target sites was performed using specific primers. For the second-step PCR amplification, PCR products from individual biological samples were amplified using different indexed primers and then pooled into sequencing samples. The samples were subsequently subjected to paired-end read sequencing using the NovaSeq-PE150 strategy at Mingma Technologies Co., Ltd. (Shanghai, China). And the data collection of next-generation sequencing was used by Illumina bcl2fastq Software (v2.20). Finally, the resultant FASTQ files were analysed using CRISPResso2^25^. The oligonucleotide sequences used for NGS are listed in Supplementary Data 4.

### Off-target analysis

For each gRNA, the off-target sites were predicted by Cas-OFFinder^23^ and CRISPOR(Version 4.99)^24^ (Supplementary Data 5). The specificity score was calculated by CRISPOR (Version 4.99)^24^ and then we chose the three most likely off-target sites (top three high-scoring sites) to assess the off-target effect through NGS.

### Statistics

Experiments were independently repeated three times. The statistical analysis was performed using the GraphPad Prism 8 software. The results are displayed as the mean value ± standard deviation (SD). Significant differences (*P* value < 0.05) between different groups were determined by a two-tailed paired *t* test. The asterisks *, ** and *** indicate significance with *P* values less than 0.05, 0.01 and 0.001, respectively. *P* values for all figures are listed in Supplementary Data 6.

### Reporting summary

Further information on research design is available in the Nature Research Reporting Summary linked to this article.

## Supplementary information

Supplementary Information

Description of Additional Supplementary Files

Supplementary Data 1

Supplementary Data 2

Supplementary Data 3

Supplementary Data 4

Supplementary Data 5

Supplementary Data 6

Reporting Summary

Peer reviewer reports

## Data Availability

The high-throughput sequencing data generated in this study have been deposited in the NCBI Sequence Read Archive database under SRA accession code PRJNA795906. All data supporting the findings of this study are available within the article and Supplementary Information files and also are available from the corresponding author upon reasonable request. Source data are provided with this paper.

## Source data

Source Data
