# Author Correction: SpG and SpRY variants expand the CRISPR toolbox for genome editing in zebrafish

**DOI:** 10.1038/s41467-023-36632-8

**Published:** 2023-02-16

**Authors:** Fang Liang, Yu Zhang, Lin Li, Yexin Yang, Ji-Feng Fei, Yanmei Liu, Wei Qin

**Affiliations:** 1 Key Laboratory of Brain, Cognition and Education Sciences, Ministry of Education; Institute for Brain Research and Rehabilitation, South China Normal University, 510631 Guangzhou, Guangdong China; 2 Institute of Modern Aquaculture Science and Engineering, Guangdong Provincial Engineering Technology Research Center for Environmentally-Friendly Aquaculture, Guangdong Provincial Key Laboratory for Healthy and Safe Aquaculture, School of Life Sciences, South China Normal University, 510631 Guangzhou, Guangdong China; 3 Department of Pathology, Guangdong Provincial People’s Hospital, Guangdong Academy of Medical Sciences, 510080 Guangzhou, Guangdong China

**Keywords:** Disease model, CRISPR-Cas9 genome editing

Correction to: *Nature Communications* 10.1038/s41467-022-31034-8, published online 14 June 2022

The original version of this Article contained an error in the Methods section “Cas9 RNP complex preparation, microinjection and image acquisition in zebrafish”, which incorrectly read ‘One-cell stage zebrafish embryos were injected with 2 nl of a solution containing 5 µM RNP complex or 300 ng/μl Cas9 mRNA and 30 ng/μl gRNA mixture. For SpRY-CBE4max or zSpRY-ABE8e mRNA, 400 ng/μl was used. For the HDR experiment, 300 ng/μl Cas9 mRNA, 30 ng/μl gRNA and 20 ng/μl donor templated were mixed and injected.’

The correct version states ‘One-cell stage zebrafish embryos were injected with 2 nl of a solution containing 5 µM RNP complex or 300 ng/μl Cas9 mRNA and 200 ng/μl gRNA mixture. For SpRY-CBE4max or zSpRY-ABE8e mRNA, 400 ng/μl was used. For the HDR experiment, 300 ng/μl Cas9 mRNA, 200 ng/μl gRNA and 20 ng/μl donor templated were mixed and injected.’

This has been corrected in both the PDF and HTML versions of the Article.

