# Supplementary material for: SpG and SpRY variants expand the CRISPR toolbox for genome editing in zebrafish
Source: Nat Commun. 2022 Jun 14;13:3421. doi: 10.1038/s41467-022-31034-8 (PMC9198057; doi:10.1038/s41467-022-31034-8)

Supplementary Information

SpG and SpRY variants expand the CRISPR toolbox for genome editing in zebrafish

Fang Liang[1, 2], Yu Zhang[1], Lin Li[1], Yexin Yang[2], Ji-Feng Fei[3], Yanmei Liu[1, *], Wei Qin[1, *]

[1] Key Laboratory of Brain, Cognition and Education Sciences, Ministry of Education; Institute for Brain Research and Rehabilitation, South China Normal University, Guangzhou, Guangdong, 510631, China.

[2] Institute of Modern Aquaculture Science and Engineering, Guangdong Provincial Engineering Technology Research Center for Environmentally-Friendly Aquaculture, Guangdong Provincial Key Laboratory for Healthy and Safe Aquaculture, School of Life Sciences, South China Normal University, Guangzhou, Guangdong, 510631, China.

[3] Department of Pathology, Guangdong Provincial People's Hospital, Guangdong Academy of Medical Sciences, Guangzhou, Guangdong, 510080, China.

Supplementary Figures

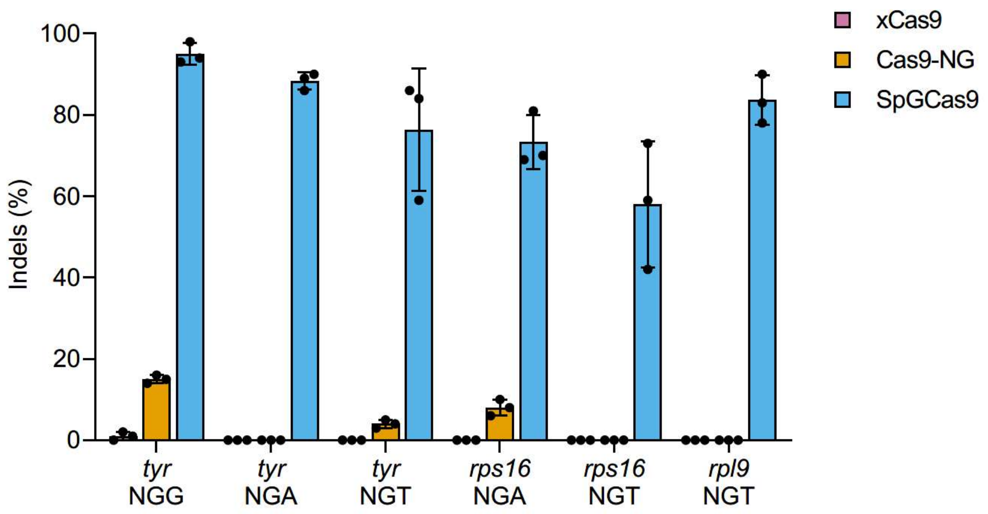

Supplementary Figure 1. Comparison of the editing efficiency of xCas9, Cas9-NG and SpGCas9 at the same sites targeting NGN PAMs in zebrafish. Editing efficiency was assessed by ICE Tools analysis (values are presented as mean value ± standard deviation (SD), *n* = 3 biological replicates). Source data are provided as a Source Data file.

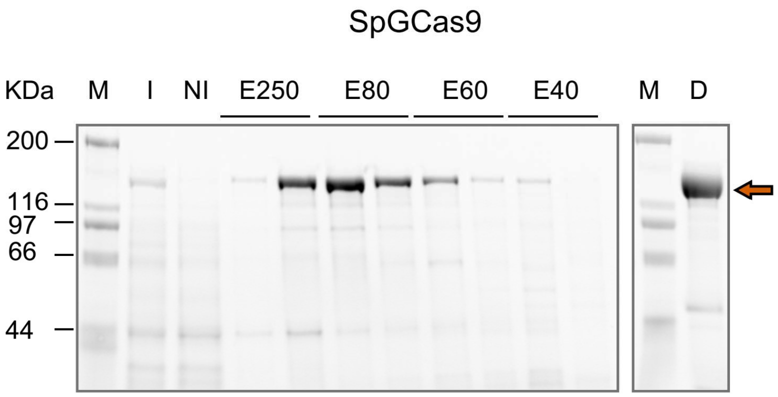

Supplementary Figure 2. SDS-PAGE gel image showing purified products of C-terminally His-tagged SpGCas9. The expression and purification of SpGCas9 were analyzed by 6% SDS-PAGE. The red arrow indicates the desired SpGCas9 protein with Ni-NTA affinity resin. M: protein marker, I: IPTG-induced, NI: noninduced control, D: purified protein after dialysis and concentration, E40~250: eluted proteins with different concentrations of imidazole, *e.g.*, E250 means elution with 250 mM imidazole. This experiment was repeated 3 times independently with similar results.

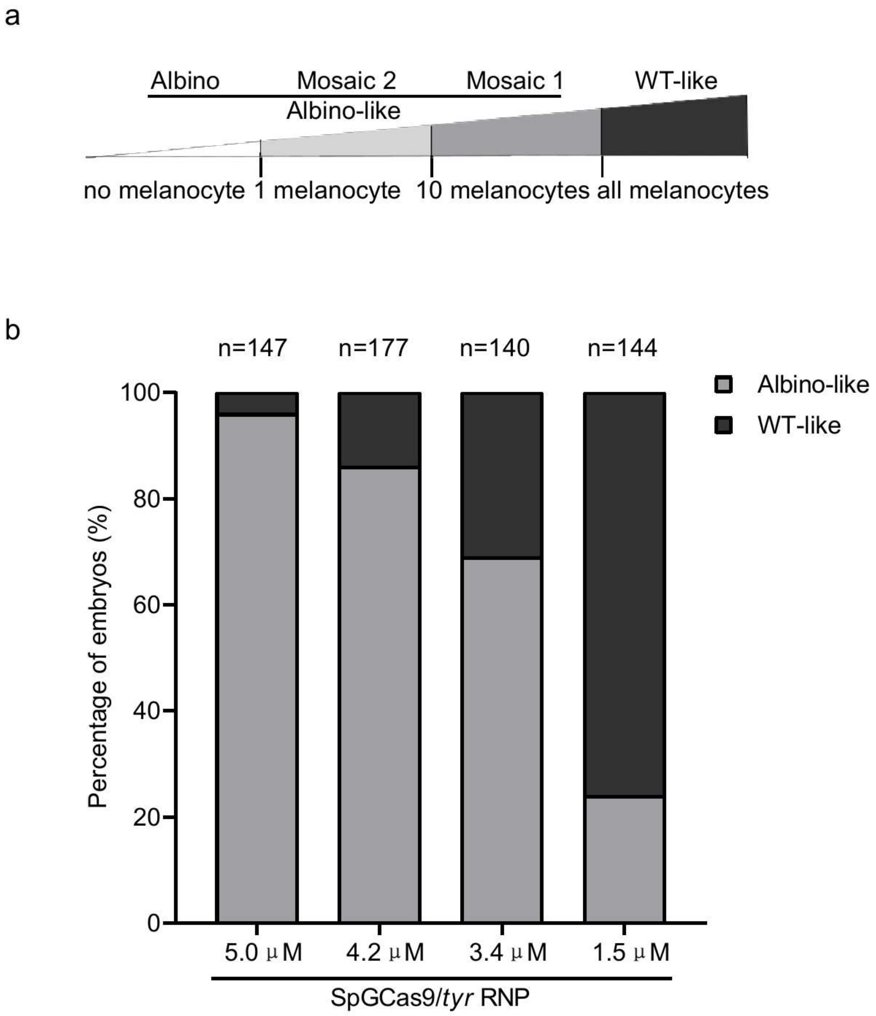

Supplementary Figure 3. Determination of the appropriate SpGCas9-gRNA RNP concentration in zebrafish. **a** Definition of mosaic pigmentation degree compared to wild-type (WT). Mosaic 1 was defined as pigmentation significantly lower than WT, but with more than 10 melanocytes. Mosaic 2 was defined as having a number of melanocytes between 1 and 10. Albino was defined as the absence of melanocytes. Albino, mosaic 2, and mosaic 1 were classified as albino-like. **b** Phenotype statistic of SpGCas9 RNP injections targeting *tyr* NGG PAM at various concentrations. The stacked column shows the percentage of albino-like (light grey) and WT-like (dark grey). Source data are provided as a Source Data file.

a

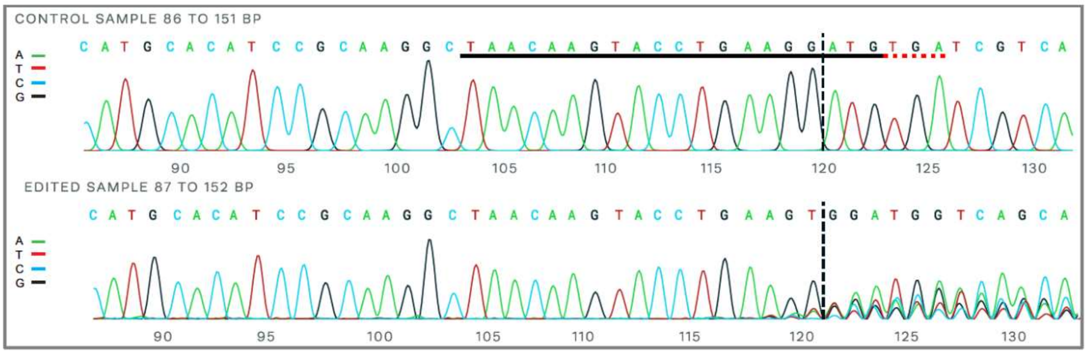

b

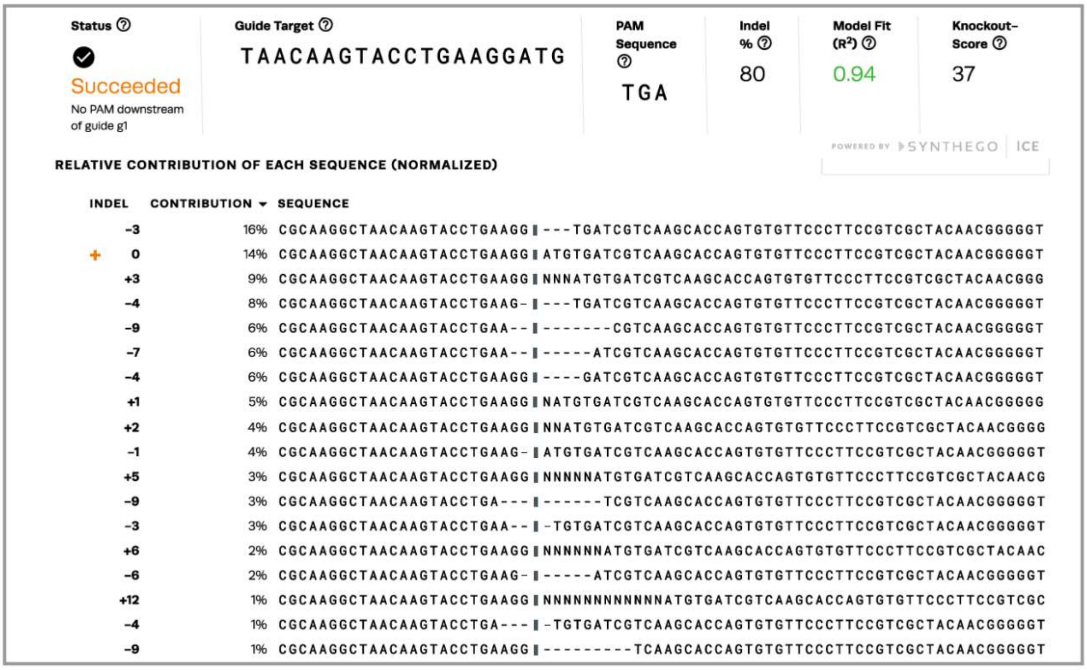

Supplementary Figure 4. The indels pattern analysis by ICE. a Sanger sequencing chromatograms of SpGCas9:*rpl17* gRNA (NGA PAM) RNP induced mutations. The black underlined sequence is the target site, and the dashed underlined TGA is PAM. The black dotted line indicates the theoretical cutting site of SpGCas9. **b** ICE analysis of SpGCas9:*rpl17* gRNA (NGA PAM) RNP induced indels in the genome.

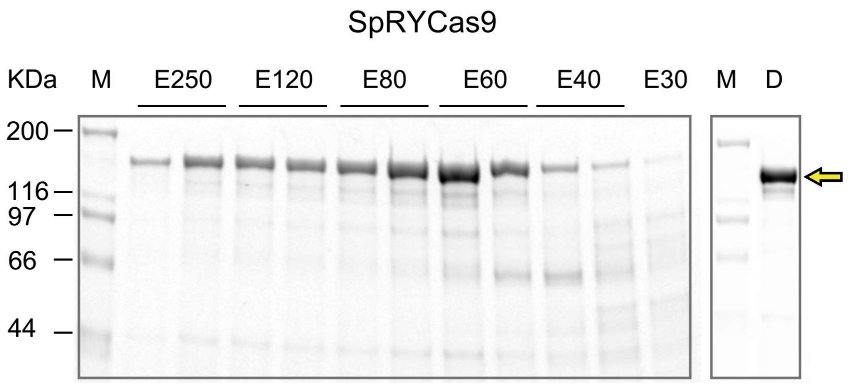

Supplementary Figure 5. SDS-PAGE gel image showing purified products of C-terminally His-tagged SpRYCas9. The purification of SpRYCas9 was analyzed by 6% SDS-PAGE. The yellow arrow indicates the purified SpRYCas9 protein. M: protein marker, D: purified protein after dialysis and concentration, E30~250: eluted proteins with different concentrations of imidazole, *e. g.* E250 means elution with 250 mM imidazole. This experiment was repeated 3 times independently with similar results.

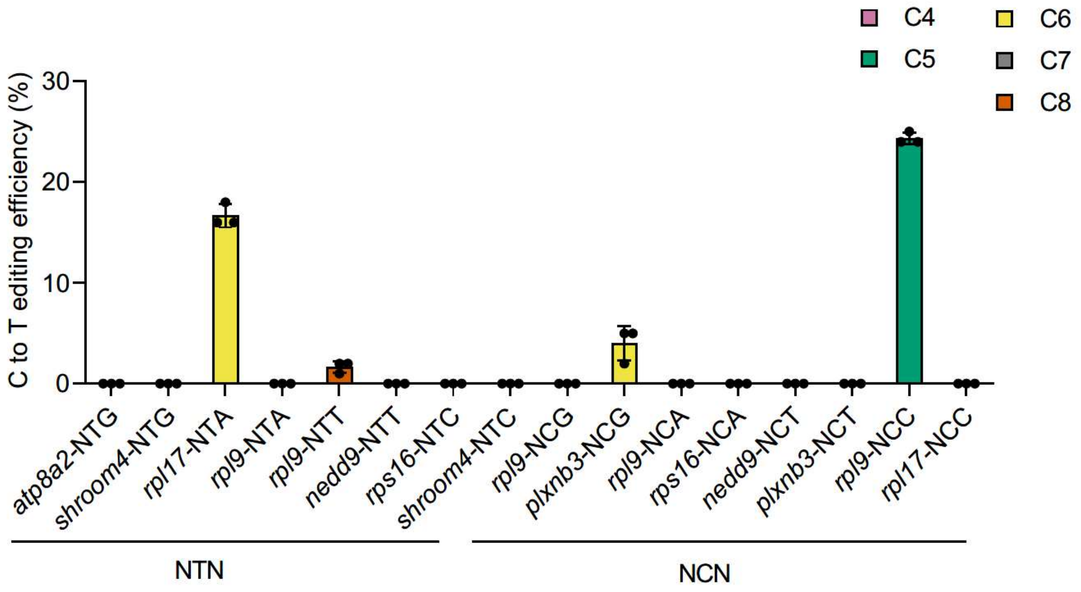

Supplementary Figure 6. Summary of C-to-T base editing efficiency of various loci with NYN PAMs induced by SpRY-CBE4max editor in zebrafish. The position of the editing base in the gRNA was labelled with numbers. (Values are presented as mean value ± standard deviation (SD), n = 3 biological replicates). Source data are provided as a Source Data file.

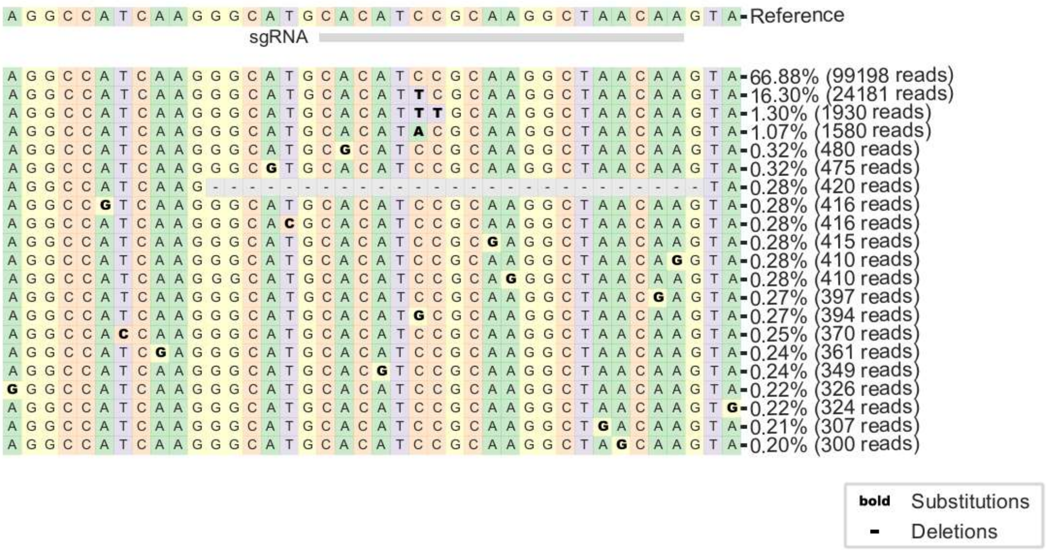

Supplementary Figure 7. On-target analysis of SpRY-CBE4max induced C-to-T editing at *rpl17*-NTA sites using NGS.

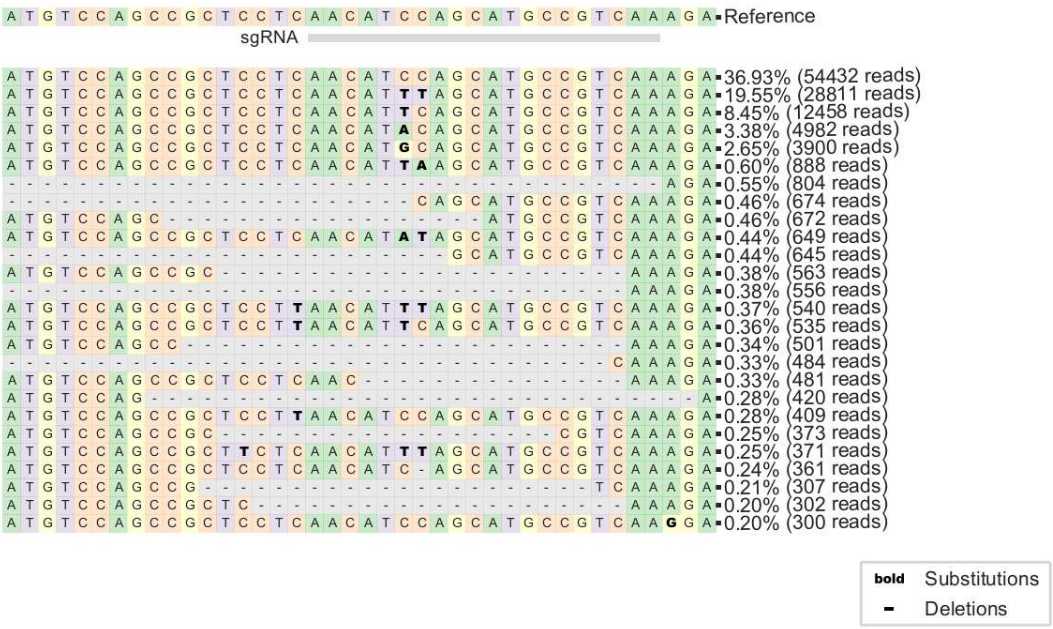

Supplementary Figure 8. On-target analysis of SpRY-CBE4max induced C-to-T editing at *ddx21*-NGA sites using NGS.

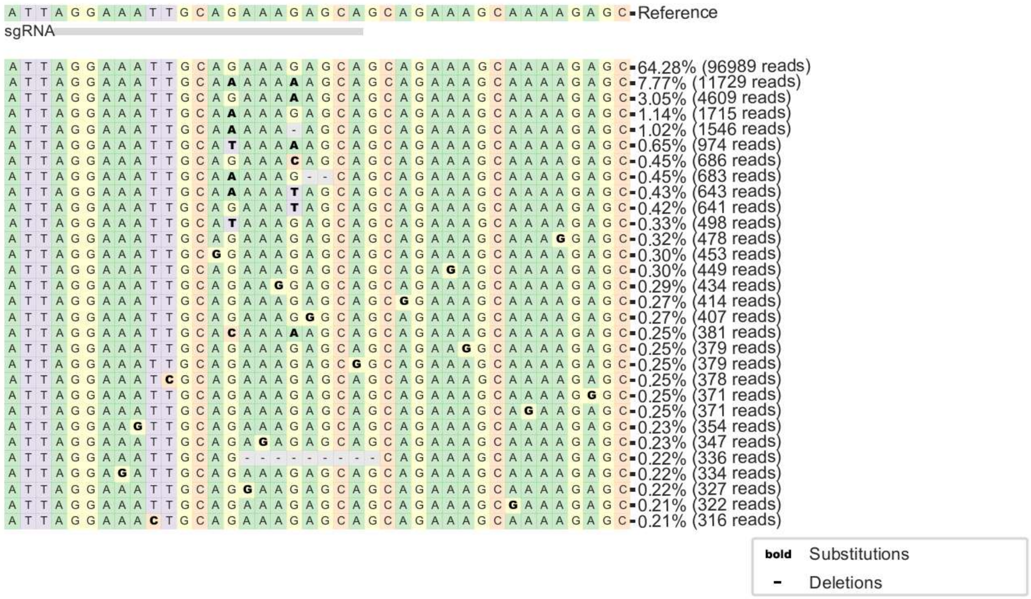

Supplementary Figure 9. On-target analysis of SpRY-CBE4max induced C-to-T editing at *mitfa*-NAT sites using NGS.

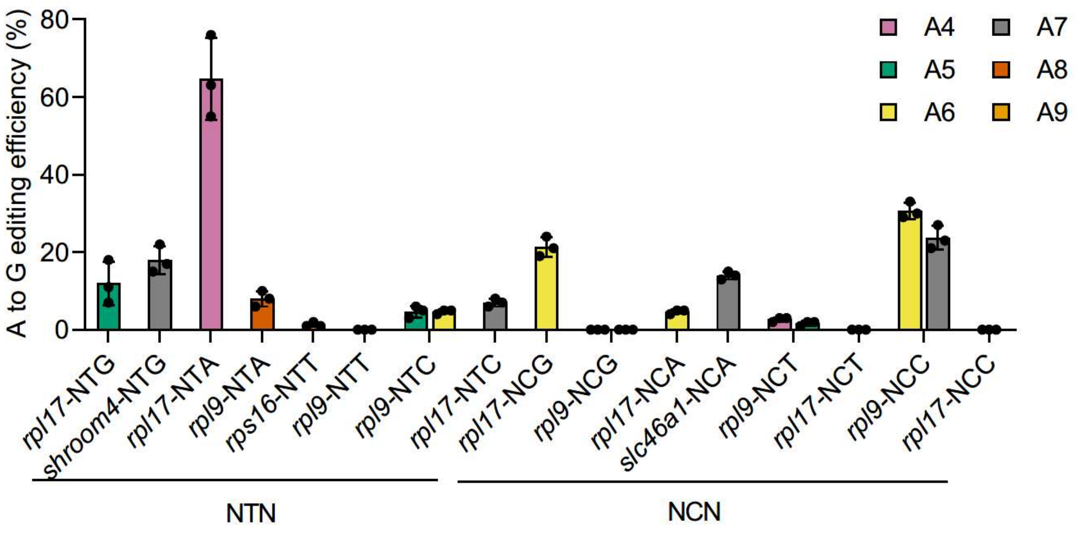

Supplementary Figure 10. Summary of A-to-G base editing efficiency of various loci with NYN PAMs induced by zSpRY-ABE8e editor in zebrafish. The position of the editing base in the gRNA was labelled with numbers. (Values are presented as mean value ± standard deviation (SD), *n* = 3 biological replicates). Source data are provided as a Source Data file.

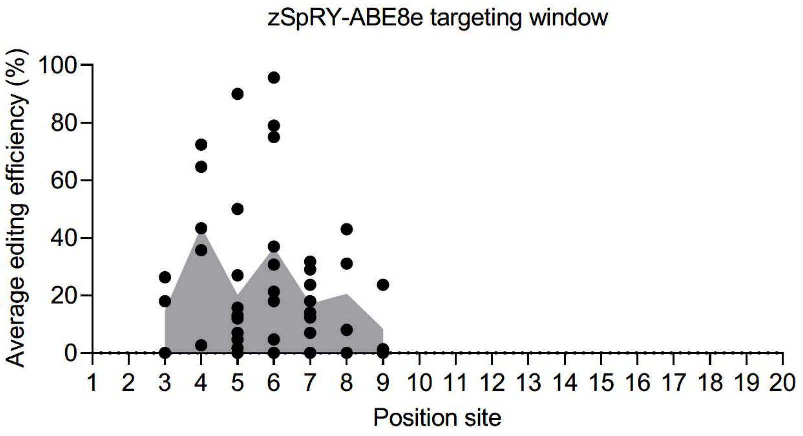

Supplementary Figure 11. Assessment of the targeting window for zSpRY-ABE8e. The targeting window of zSpRY-ABE8e was shadowed in grey (from position 3 to 9 counting from 5' terminal to 3' terminal of targeting site). Each data point represents the averaged editing activity at the particular site. Source data are provided as a Source Data file.

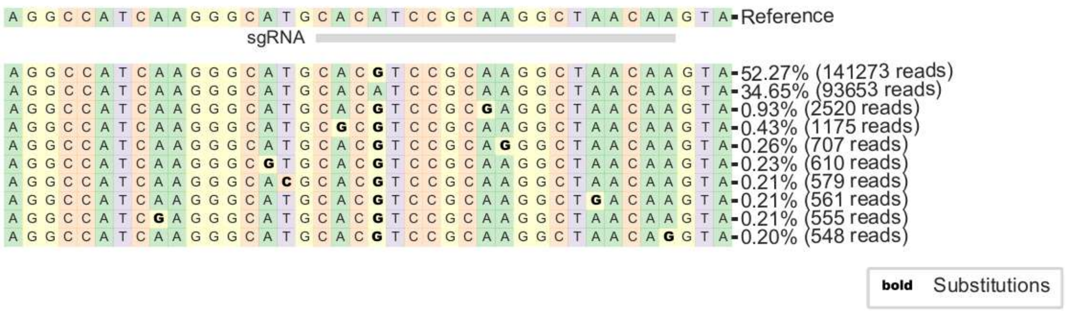

Supplementary Figure 12. On-target analysis of zSpRY-ABE8e induced A-to-G editing at *rpl17*-NTA sites using NGS.

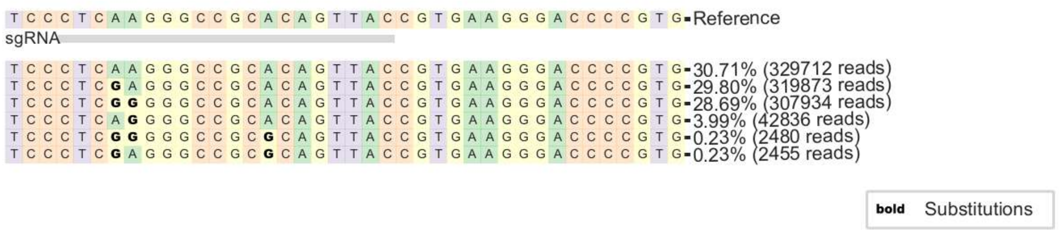

Supplementary Figure 13. On-target analysis of zSpRY-ABE8e induced A-to-G editing at *rpl9*-NGT sites using NGS.

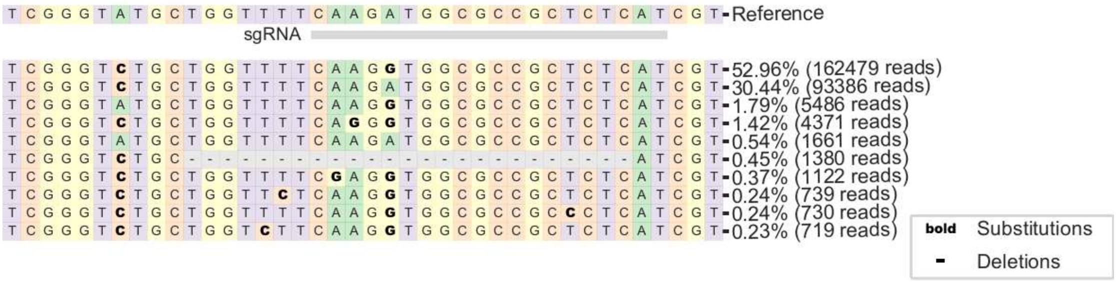

Supplementary Figure 14. On-target analysis of zSpRY-ABE8e induced A-to-G editing at *tsr2*-NGT sites using NGS.

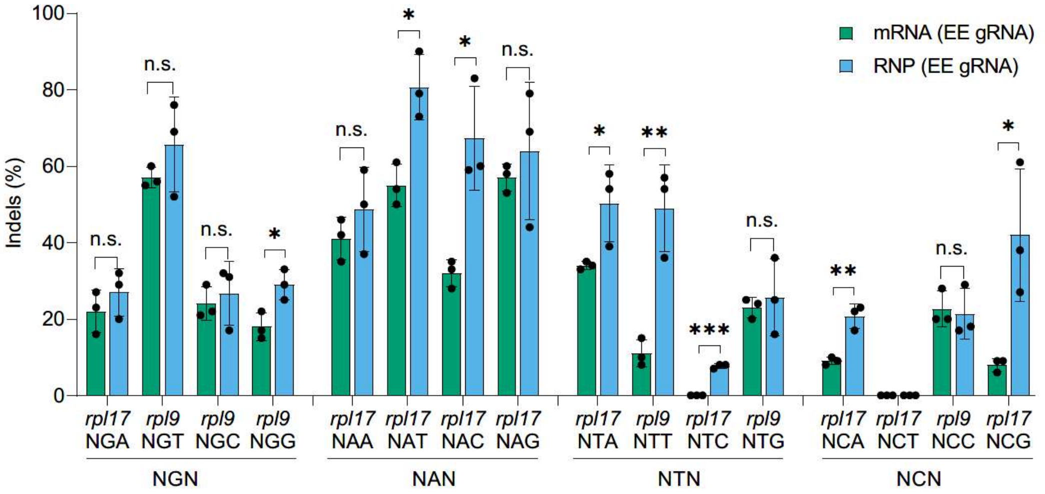

Supplementary Figure 15. Comparison of the editing efficiency of SpRY protein and mRNA with EE gRNA at the same sites targeting NNN PAMs in zebrafish. Editing efficiency was assessed by ICE Tools analysis. (Values are presented as mean value ± standard deviation (SD), n = 3 biological replicates). Data were analysed by two-tailed paired t-test. * $P \leqslant 0.05$, ** $P \leqslant 0.01$ and *** $P \leqslant 0.001$ (n.s. not significant). The exact P values are listed in Supplementary data 6. Source data are provided as a Source Data file.

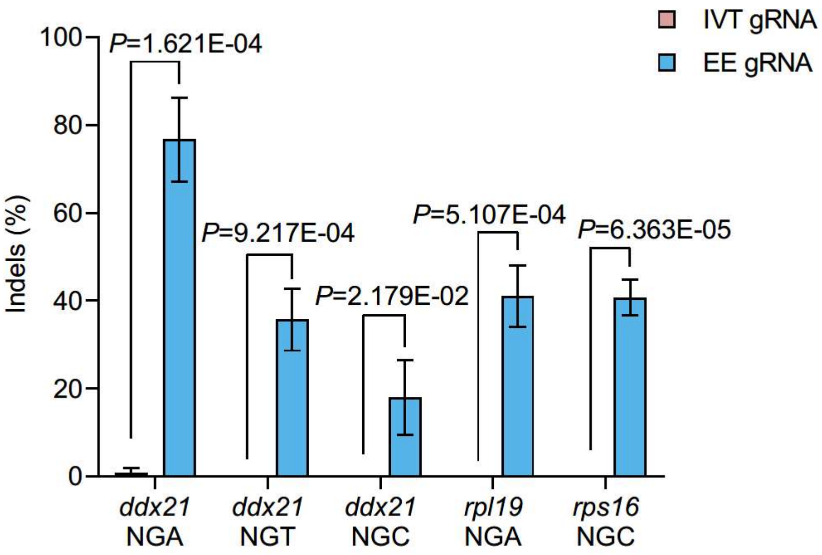

Supplementary Figure 16. MS modified gRNAs showing significantly higher targeting efficiency than the IVT gRNAs in the 5 loci of zebrafish. (Values are presented as mean value ± standard deviation (SD), *n* = 3 biological replicates). Two-tailed paired *t*-test were performed (with *P* values marked). Source data are provided as a Source Data file.

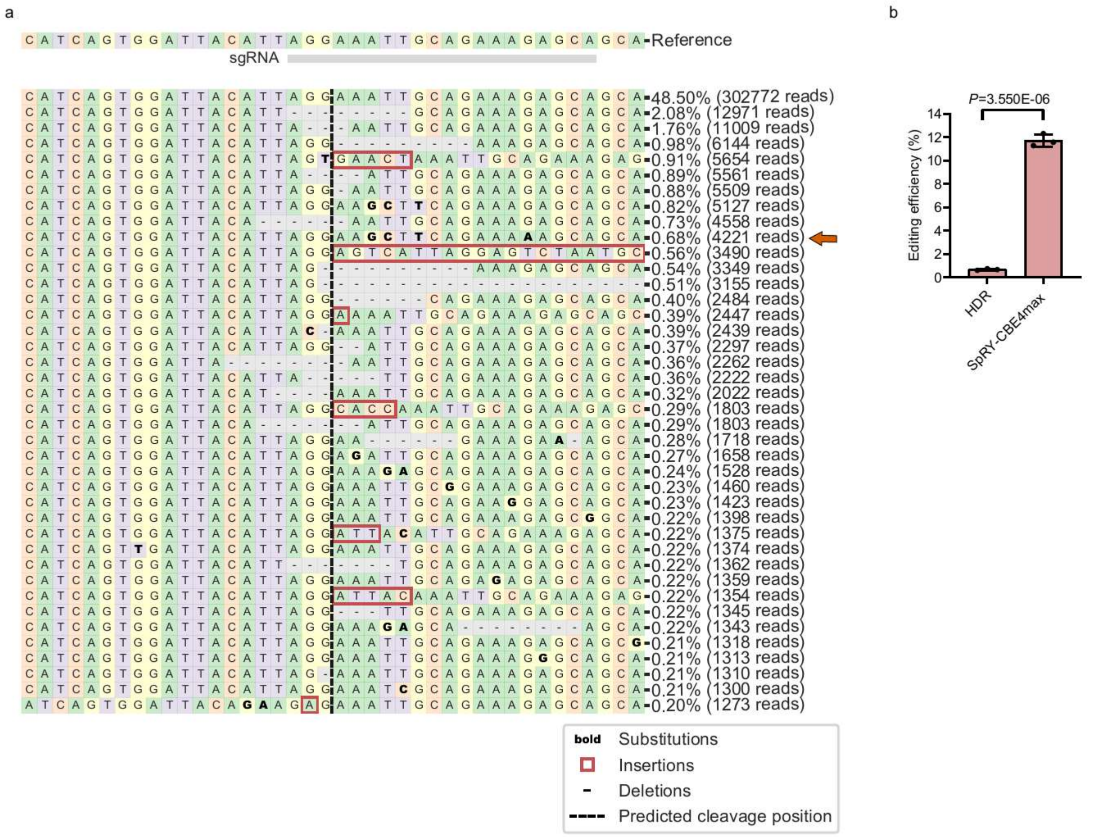

Supplementary Figure 17. NGS analysis of precise point mutation introduction to *mitfa*. a Analysis of HDR-induced C-to-T editing at *mitfa*. The red arrow indicated the correct HDR reads. **b** Bar plot comparison of HDR- or SpRY-CBE4max-induced C-to-T editing at *mitfa* sites. (values are presented as mean value ± standard deviation (SD), n = 3 biological replicates). Two-tailed paired t-test were performed (with P values marked). Source data are provided as a Source Data file.

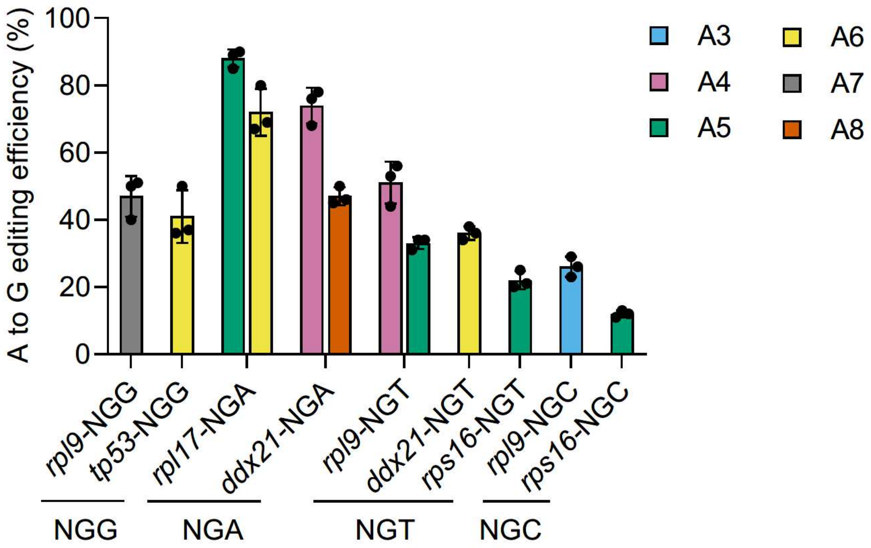

Supplementary Figure 18. Summary of A-to-G base editing efficiency of various loci with NGN PAMs induced by zSpG-ABE8e editor in zebrafish. The position of editing base in the gRNA was labelled with numbers. (Values are presented as mean value ± standard deviation (SD), *n* = 3 biological replicates). Source data are provided as a Source Data file.

Supplementary Tables

Supplementary Table 1. Off-target analysis of SpG nuclease.

gRNA	Off-target site	Mismatched bases(bp)	Genome location	Sequence (N20+PAM)	Efficiency (reads)
rpl17-NGA	1	3	8:30668379	TAAtAAGaAaCTGAAGGATGAGG	0.50% (707/141401)
	2	3	22:3883532	TAAtAAtTAtCTGAAGGATGAGC	0.48% (532/110817)
	3	3	20:20939945	aAACAtGTACaTGAAGGATGTGT	0% (0/142580)
rpl9-NGT	1	3	21:14193066	CTCAAtGGCaGCACAGTgACAGT	0% (0/136601)
	2	3	11:5459995	CTCAAGGGCCaCACAGTagCAGG	0% (0/118727)
	3	3	23:32000757	CTCAAGGaCtGCAgAGTTACAGC	0% (0/140651)
ddx21-NGC	1	2	20:29906243	CCGCTCtTCgACATCCAGCATGG	0% (0/152282)
	2	3	10:10992759	CCtCTCCTCttCATCCAGCATGT	0% (0/123749)
	3	2	11:30494286	CCcCTCCTCAACATCCAGaATGT	0% (0/132346)

Supplementary Table 2. Germline targeting efficiency and germline transmission rate of SpG and SpRY-induced indels in zebrafish

Gene	Germline targeting efficiency	Germline transmission rate
tyr-NGT	100.0% (6/6)	3#, 62.5% ((15/24))
ddx21-NGC	100.0% (11/11)	5#, 41.7% (10/24)
ddx21-NGT	100.0% (3/3)	1#, 50.0% (12/24)
rpl17-NAT	80.0% (8/10)	8#, 33.3% (8/24)

Supplementary Table 3. Germline targeting efficiency and germline transmission rate of zSpRY-ABE8e and SpRY-CBE4max.

Base editor	Gene	Germline targeting efficiency	Germline transmission rate
zSpRY-ABE8e	*rpl9*-NGT	80.0% (4/5)	1#, 66.7% (16/24)
	rpl17-NCG	75.0% (3/4)	3#, 60.9% (14/23)
	tsr2	33.3% (1/3)	2#, 55.0% (11/20)
SpRY-CBE4max	*rpl17*-NTA	66.7% (2/3)	1#, 62.5% (15/24)
	rpl9-NCC	100.0% (2/2)	1#, 43.5% (10/23)

Supplementary Data 1: The relationship between nuclease-induced indels efficiency and phenotype observed in F0 embryos. This dataset is provided in a separate “xlsx” file.

Supplementary Data 2: Primers for detection and mutations in this study. This dataset is provided in a separate “xlsx” file.

Supplementary Data 3: All target sites used in this study. This dataset is provided in a separate “xlsx” file.

Supplementary Data 4: Primers for NGS in this study. This dataset is provided in a separate “xlsx” file.

Supplementary Data 5: The predicted off-target sites in this study. This dataset is provided in a separate “xlsx” file.

Supplementary Data 6: P values calculated in the study. This dataset is provided in a separate “xlsx” file.

The raw blots and gels in Fig. S2.

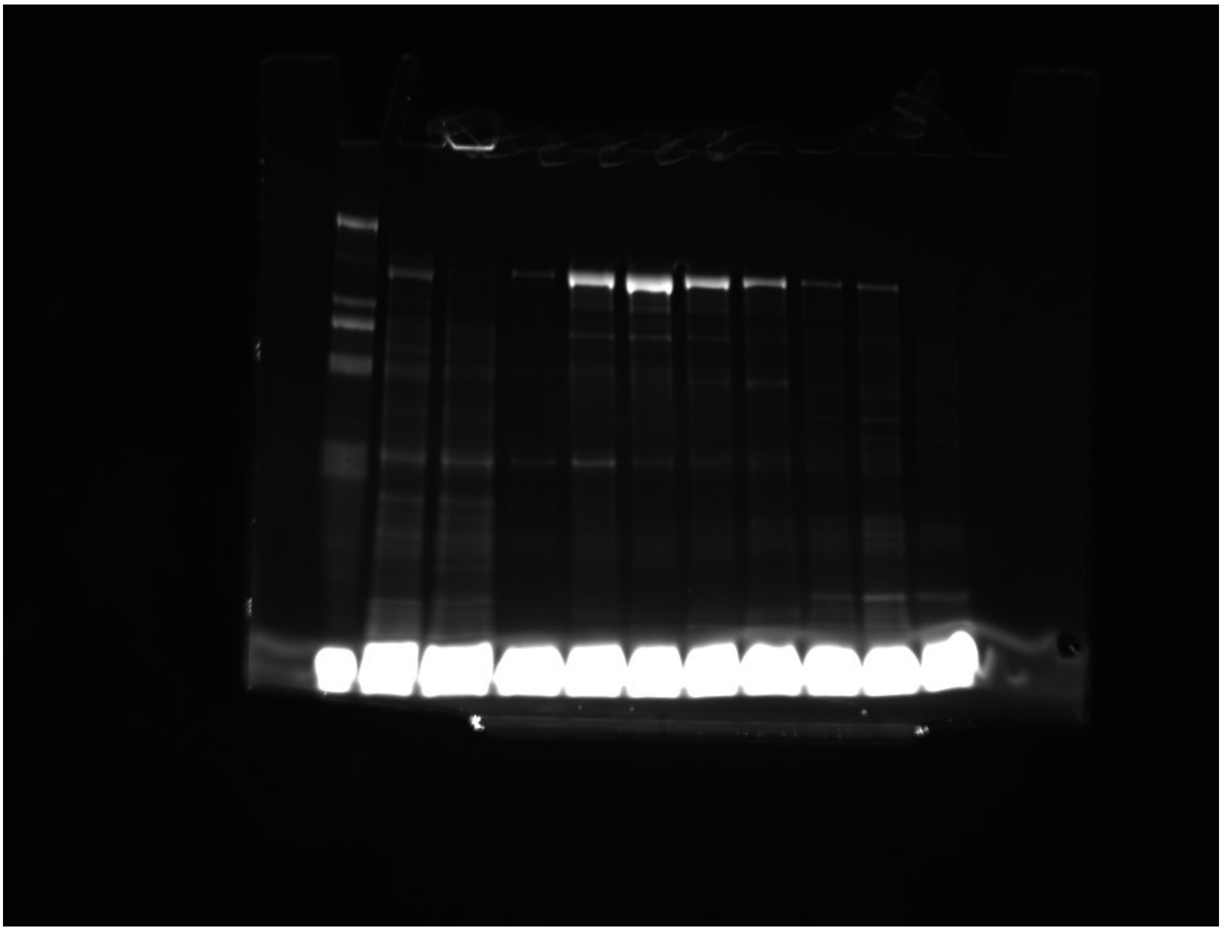

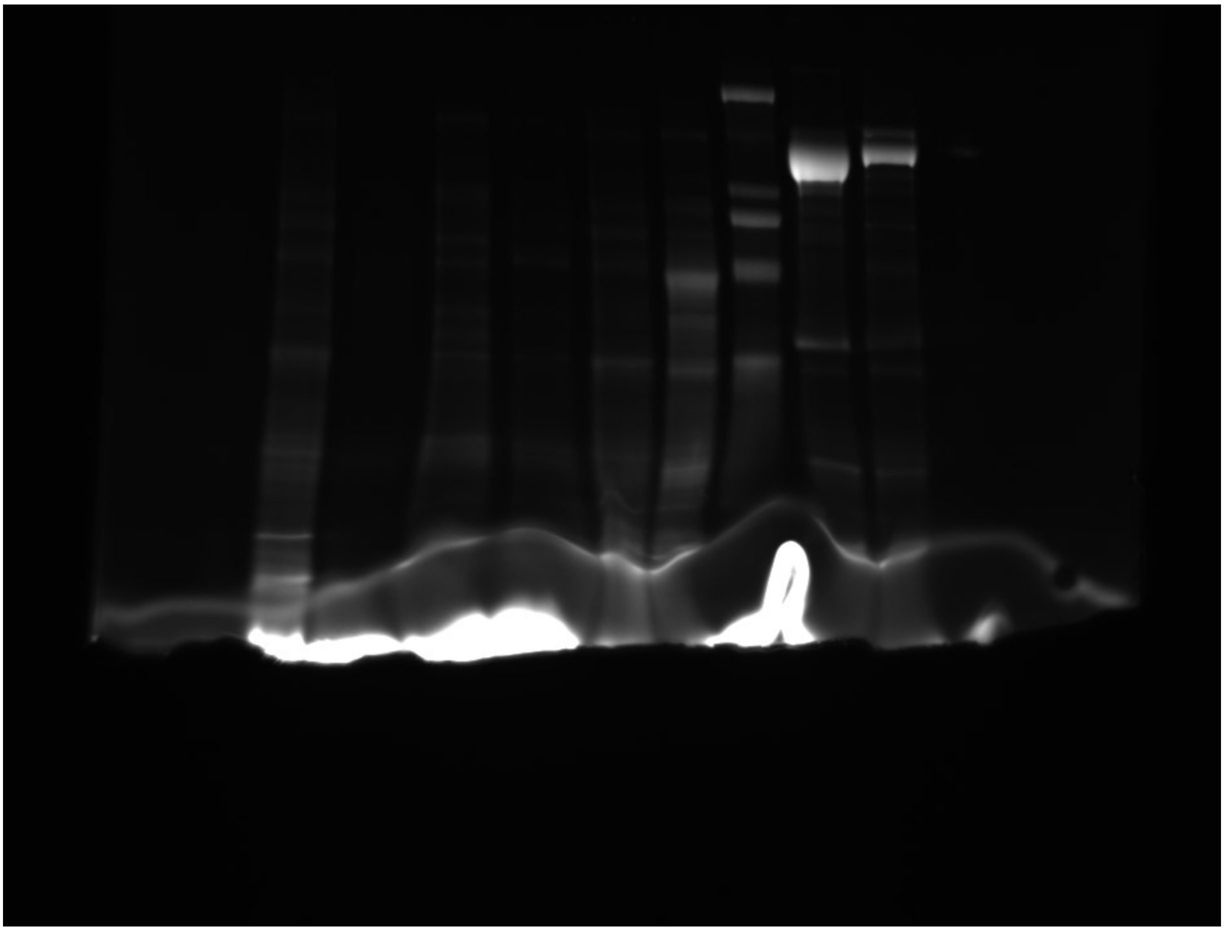

The raw blots and gels in Fig. S5.

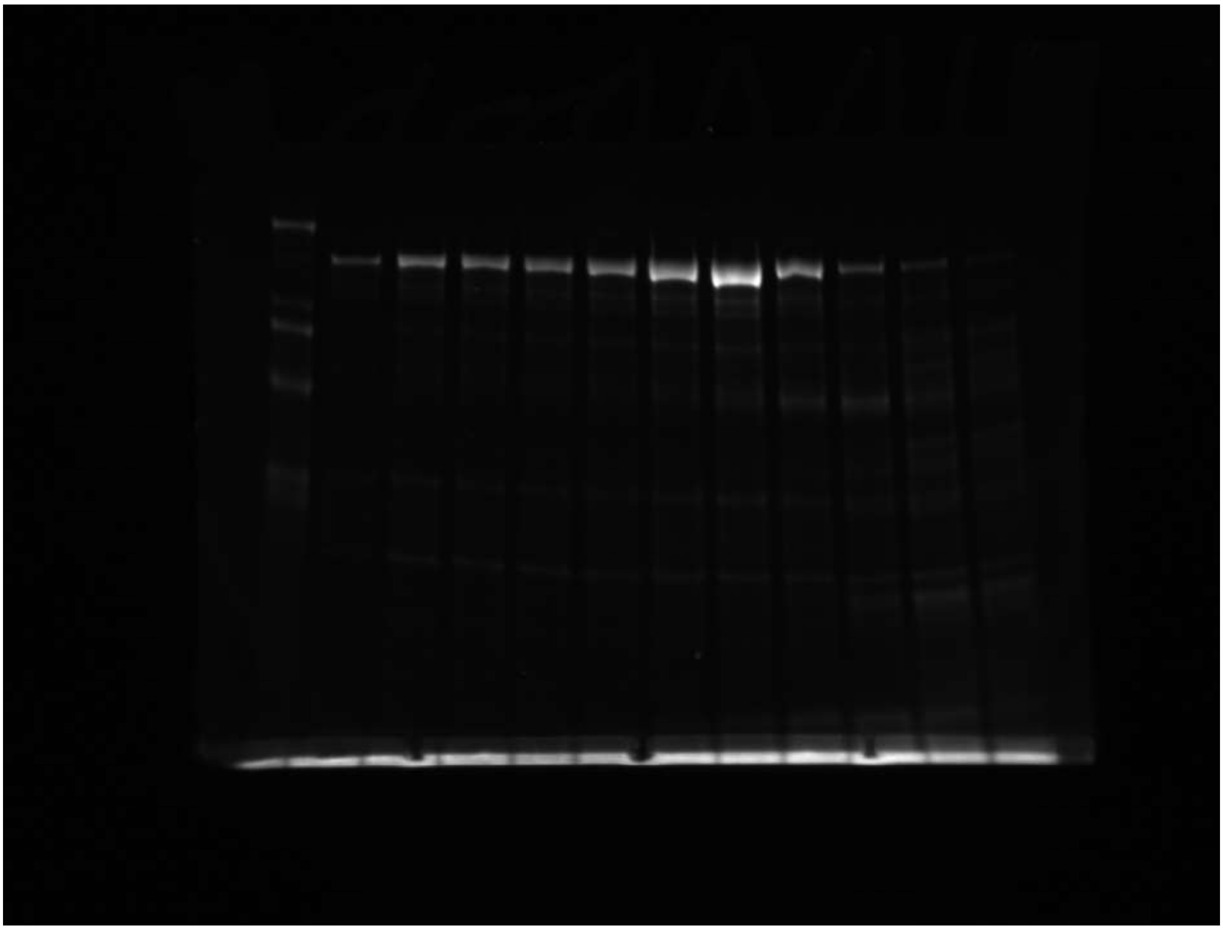

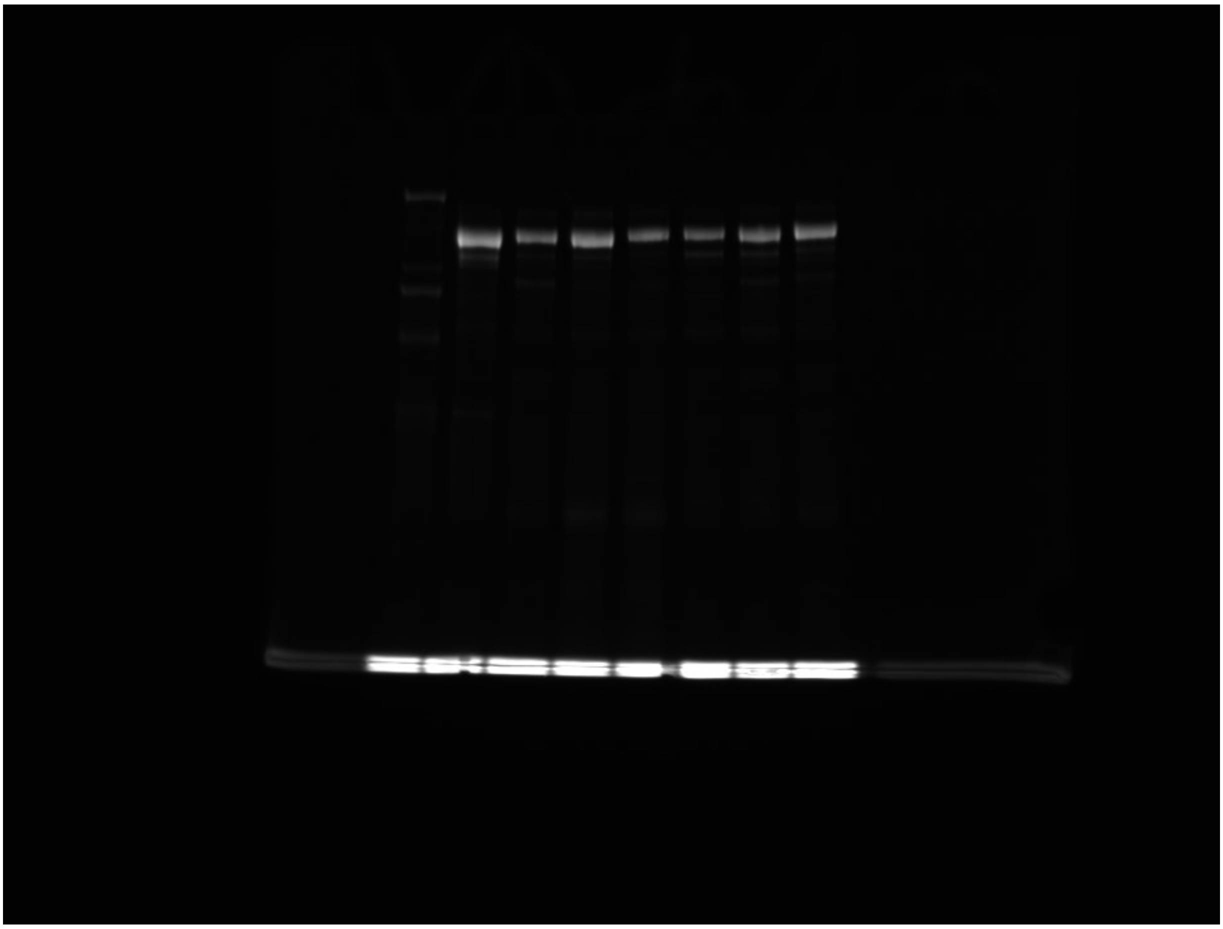

Supplement: Supplementary file 1 — Supplementary Information [file 41467_2022_31034_MOESM1_ESM.pdf]
