# Supplementary material for: SpG and SpRY variants expand the CRISPR toolbox for genome editing in zebrafish
Source: Nat Commun. 2022 Jun 14;13:3421. doi: 10.1038/s41467-022-31034-8 (PMC9198057; doi:10.1038/s41467-022-31034-8)
Supplement: Supplementary file 2 — Description of Additional Supplementary Files [file 41467_2022_31034_MOESM2_ESM.pdf]

Title: Supplementary Data 1

Description: The relationship between nuclease-induced indels efficiency and phenotype observed in F0 embryos.

Title: Supplementary Data 2

Description: Primers for detection and mutations in this study.

Title: Supplementary Data 3

Description: All target sites used in this study.

Title: Supplementary Data 4

Description: Primers for NGS in this study.

Title: Supplementary Data 5

Description: The predicted off-target sites in this study.

Title: Supplementary Data 6

Description: *P* values calculated in the study.
