# Supplementary material for: SpG and SpRY variants expand the CRISPR toolbox for genome editing in zebrafish
Source: Nat Commun. 2022 Jun 14;13:3421. doi: 10.1038/s41467-022-31034-8 (PMC9198057; doi:10.1038/s41467-022-31034-8)
Supplement: Supplementary file 9 — Reporting Summary [file 41467_2022_31034_MOESM9_ESM.pdf]

nature portfolio

Corresponding author(s): Wei Qin and Yanmei Liu

Last updated by author(s): May 24, 2022

Reporting Summary

Nature Portfolio wishes to improve the reproducibility of the work that we publish. This form provides structure for consistency and transparency in reporting. For further information on Nature Portfolio policies, see our Editorial Policies and the Editorial Policy Checklist.

Statistics

For all statistical analyses, confirm that the following items are present in the figure legend, table legend, main text, or Methods section.

n/a	Confirmed	
☐	☒	The exact sample size (*n*) for each experimental group/condition, given as a discrete number and unit of measurement
☐	☒	A statement on whether measurements were taken from distinct samples or whether the same sample was measured repeatedly
☐	☒	The statistical test(s) used AND whether they are one- or two-sided<br>*Only common tests should be described solely by name; describe more complex techniques in the Methods section.*
☒	☐	A description of all covariates tested
☒	☐	A description of any assumptions or corrections, such as tests of normality and adjustment for multiple comparisons
☐	☒	A full description of the statistical parameters including central tendency (e.g. means) or other basic estimates (e.g. regression coefficient) AND variation (e.g. standard deviation) or associated estimates of uncertainty (e.g. confidence intervals)
☐	☒	For null hypothesis testing, the test statistic (e.g. *F*, *t*, *r*) with confidence intervals, effect sizes, degrees of freedom and *P* value noted<br>*Give P values as exact values whenever suitable.*
☒	☐	For Bayesian analysis, information on the choice of priors and Markov chain Monte Carlo settings
☒	☐	For hierarchical and complex designs, identification of the appropriate level for tests and full reporting of outcomes
☒	☐	Estimates of effect sizes (e.g. Cohen's *d*, Pearson's *r*), indicating how they were calculated

Our web collection on statistics for biologists contains articles on many of the points above.

Software and code

Policy information about availability of computer code

Data collection	Images were taken by SZX2-FOF microscope (OLYMPUS) with either an XM10 digital camera (OLYMPUS) or AxioCam MRc5 digital camera (Leica). For NGS data, the samples were subsequently subjected to paired-end read sequencing using the NovaSeq-PE150 strategy at Mingma Technologies Co., Ltd. (Shanghai, China). And the data collection of next-generation sequencing was used by Illumina bcl2fastq Software (v2.20)
Data analysis	ICE website (Synthego Co.) was used to analyze the indel mutation frequency.<br>Base editing results were analyzed by EditR (1.0.10) program. Kluesner M, Nedveck D, Lahr W, Moriarity B. EditR: A method to quantify base editing via Sanger sequencing. The CRISPR Journal. 2018.<br>NGS results were analyzed by CRISPResso2. Clement K, Rees H, Canver MC, Gehrke JM, Farouni R, Hsu JY, et al. CRISPResso2 provides accurate and rapid genome editing sequence analysis. Nature biotechnology 2019, 37(3): 224-226.<br>The off-target sites were predicted by Cas-OFFinder and CRISPOR (Version 4.99). The specificity score was calculated by CRISPOR (Version 4.99).<br>Adobe Photoshop CC software,GraphPad Prism 8 software

For manuscripts utilizing custom algorithms or software that are central to the research but not yet described in published literature, software must be made available to editors and reviewers. We strongly encourage code deposition in a community repository (e.g. GitHub). See the Nature Portfolio guidelines for submitting code & software for further information.

Data

Policy information about availability of data

All manuscripts must include a data availability statement. This statement should provide the following information, where applicable:

- Accession codes, unique identifiers, or web links for publicly available datasets
- A description of any restrictions on data availability
- For clinical datasets or third party data, please ensure that the statement adheres to our policy

High-throughput sequencing data can be accessed via NCBI Sequence Read Archive database with SRA accession code PRJNA795906. All data supporting the findings of this study are available within the article and Supplementary information files, and also are available from the corresponding author upon reasonable request. Source data are provided with this paper.

Field-specific reporting

Please select the one below that is the best fit for your research. If you are not sure, read the appropriate sections before making your selection.

- [x] Life sciences
- [ ] Behavioural & social sciences
- [ ] Ecological, evolutionary & environmental sciences

For a reference copy of the document with all sections, see nature.com/documents/nr-reporting-summary-flat.pdf

Life sciences study design

All studies must disclose on these points even when the disclosure is negative.

Sample size	No statistical methods were used to predetermine sample size for zebrafish experiments.
Data exclusions	No data were excluded.
Replication	Reported results were replicated three times. All attempts at replication were successfull.
Randomization	Zebrafish in-crossing was performed by random selection of mating pairs. The allocation of zebrafish into control or editing group was done at random.
Blinding	Zebrafish husbandry was performed under identical conditions, blinding was not used. Blinding was not necessary in our quantitative experiments.

Reporting for specific materials, systems and methods

We require information from authors about some types of materials, experimental systems and methods used in many studies. Here, indicate whether each material, system or method listed is relevant to your study. If you are not sure if a list item applies to your research, read the appropriate section before selecting a response.

Materials & experimental systems

n/a	Involved in the study	
x		Antibodies
x		Eukaryotic cell lines
x		Palaeontology and archaeology
	x	Animals and other organisms
x		Human research participants
x		Clinical data
x		Dual use research of concern

Methods

n/a	Involved in the study	
x		ChIP-seq
x		Flow cytometry
x		MRI-based neuroimaging

Animals and other organisms

Policy information about studies involving animals; ARRIVE guidelines recommended for reporting animal research

Laboratory animals	Wild-type AB line embryos were raised under standard conditions. The selection of mating pairs (12-15 months) was random from a pool of 30 males and 30 females.
Wild animals	The study did not involve wild animals.
Field-collected samples	The study did not involve samples collected from the field.

Ethics oversight	All animal protocols were reviewed and approved by the University Animal Care and Use Committee of the South China Normal University.

Note that full information on the approval of the study protocol must also be provided in the manuscript.
