# Supplementary material for: SpG and SpRY variants expand the CRISPR toolbox for genome editing in zebrafish
Source: Nat Commun. 2022 Jun 14;13:3421. doi: 10.1038/s41467-022-31034-8 (PMC9198057; doi:10.1038/s41467-022-31034-8)
Supplement: Supplementary file 10 — Peer reviewer reports [file 41467_2022_31034_MOESM10_ESM.pdf]

nature portfolio

Peer Review File

SpG and SpRY variants expand the CRISPR toolbox for genome editing in zebrafish

Open Access This file is licensed under a Creative Commons Attribution 4.0 International License, which permits use, sharing, adaptation, distribution and reproduction in any medium or format, as long as you give appropriate credit to the original author(s) and the source, provide a link to the Creative Commons license, and indicate if changes were made. In the cases where the authors are anonymous, such as is the case for the reports of anonymous peer reviewers, author attribution should be to 'Anonymous Referee' followed by a clear attribution to the source work. The images or other third party material in this file are included in the article's Creative Commons license, unless indicated otherwise in a credit line to the material. If material is not included in the article's Creative Commons license and your intended use is not permitted by statutory regulation or exceeds the permitted use, you will need to obtain permission directly from the copyright holder. To view a copy of this license, visit http://creativecommons.org/licenses/by/4.0/.

Reviewers' Comments:

Reviewer #1:
Remarks to the Author:
Liang et al describe a CRISPR/Cas9-based genome editing tool in zebrafish using SpG and SpRY Cas9 variants. These two variants can target NGN or NNN PAM sites. SpRY variant was further fused with ABE and CBE base editors to edit specific bases on a target site. Overall, tools described in this manuscript could be helpful to the zebrafish community. However, several questions need to be addressed, specifically:

Page 2:
1. Authors state x-Cas9 could not edit the zebrafish genome (unpublished data). However, it is not true, I am aware of few zebrafish labs using x-Cas9 on NGT and similar PAM sites, albeit at lower rates. Would you please provide data to support this claim?
2. Cas9-NG has been shown to edit the genome at a higher rate than the x-Cas9 in several species. Did the authors compare the activities of Cas9-NG and SpG?
3. The data presented on tyr targeting on canonical site is surprising (Figure 1C) given several groups have shown almost 100% efficiency with protein and 80-90% with mRNA in generating albino animals. Therefore, I would have expected a significant improvement between mRNA and protein.
4. Add a reference to SpCas9 mRNA and protein comparison.
5. Authors show SpG can edit the genome at NGA and NGG PAM with variable efficiencies using RNP, and it is surprising that no activities were detected using in vitro synthesized guides on non-canonical PAM. This data is also contradicting previously published data in a preprint by the Moreno-Mateos group. This preprint has demonstrated the use of IVT guides at various PAMs as recognized by SpG Cas9 or SpRY. Probably, testing some of those guides will be useful.
6. Mutagenesis activities were measured by Sanger sequencing followed by ICE indel analysis. ICE tool does not recognize the non-canonical PAMs. Therefore, I believe the mutagenesis efficiencies are not accurate. I would suggest either performing next-gen sequencing or clonal sequencing if authors choose to use Sanger sequencing.
7. Did the authors determine the base editing activities using SpG variant?
8. CBE4max is not the most specific base editors, as shown in this manuscript as well. There are many bystander mutations outside of the editing window; therefore, calling it very specific is not supported by the data.
9. How many embryos showed pigment phenotypes at 2dpf following by base editing?
10. The authors claimed no indels were detected in base editing experiments, but this conclusion is based on Sanger Sequencing of injected embryos. Deep sequencing might shed more light on the specificity of the base editor.
11. Similarly, Sanger sequencing cannot determine whether the ABE8e system can generate high product purity in zebrafish.
12. In Table S1, around ten targets showed 0% editing. Does this mean either the first base or third base in the PAM influences the editing activities?
13. Did the authors compare the activities of the base editor using RNP vs mRNA? This experiment is essential given higher efficiencies will allow us to test disease-causing variants rapidly.

Minor point:

Please change CRISPR-Cpf1 to CRISPR/Cas12a.

Reviewer #2:
Remarks to the Author:
Notes on: SpG and SpRY variants expand the CRISPR toolbox for genome editing in zebrafish

Remarks to the Author:
In this work, Liang et al. use the recently described SpG and SpRY Cas9 as nucleases and base editor (CBE and ABE) fusions and demonstrate high efficiency on-target editing and high product purity across a wide variety of different PAM sequences thereby demonstrating the usability of

these novel PAM-flexible Cas9 variants in zebrafish. Using these tools, the authors were able to generate previously inaccessible genetic variants. Even though the presented results are important, especially the data on SpRY base editing (Figure 3 and 4) should be much more comprehensive both regarding the number of investigated target sites and the presentation of the data. Additionally, important aspects that come to mind when using PAM-flexible Cas9 variants such as the putatively increased risk for off-target editing have not been investigated by the authors. Furthermore, the use of NGS and a more thorough comparison to existing technology would be necessary to make this study of interest to a broader readership.

Major comments:

1) Fig 2) It appears that only one spacer is used to assess the impact of a specific PAM sequence. The spacer sequence could be a big confounder on the efficiencies reported in Fig. 2d. The authors should perform this experiment with more spacers e.g. three per particular PAM sequence to be able to draw conclusion on the influence of a particular PAM or alternatively only present the data in collapsed form (Fig. 2e).
2) Fig 3) and 4) Instead of showing representative sanger traces, the authors should show barplots analogous to Fig 1) and Fig2) that allow the reader to assess the variance of gene editing. The authors should also elaborate on why they have chosen a different delivery modality for the base editors as compared to nucleases.
3) The authors should include more target sites with a greater diversity of PAM sequences in their assessment of SpRY CBE and ABE gene editing efficiencies.
4) The authors should elaborate on the implications of their results in the context of the recently described uses of prime editing in zebrafish.
5) An analysis of potential off-target effects of SpRY nuclease and base editors is missing. It would be great if the authors could analyse the degree to which off-targets occur with the analysed PAM-flexible Cas9 variants since the increased PAM-flexibility might also increase the risk for off-target gene editing.
6) It would be great if the authors could compare SpRY to other PAM-flexible variants that have been recently described (e.g. Nishimasu et al., Science, 2018).
7) It would be advisable to perform NGS on at least some of the base edited samples to comprehensively assess the purity of base editing products.
8) It could be helpful if the authors would compare SpRY CBE and ABE base editing to SpRY-mediated HDR in terms of editing efficiency and product purity. The authors should then give recommendations on which technology to use in which cases.

Minor comments:

1) Individual data points should be shown for all figures.
2) Fig 1d. is not a histogram as stated in the figure legend.

Response to Reviewers

We thank the reviewers and editor for constructive critiques. We revised the manuscript accordingly. The following specific answers to each question should also address concerns summarized by the editors. We think the paper is improved and hope it is acceptable for publication in Nature Communications. A copy of track changes is also included.

REVIEWER COMMENTS

Reviewer #1 (Remarks to the Author):

Liang et al describe a CRISPR/Cas9-based genome editing tool in zebrafish using SpG and SpRY Cas9 variants. These two variants can target NGN or NNN PAM sites. SpRY variant was further fused with ABE and CBE base editors to edit specific bases on a target site. Overall, tools described in this manuscript could be helpful to the zebrafish community. However, several questions need to be addressed, specifically:

Response: We truly thank the reviewer for the positive feedback and highly appreciate the recognition of the significance of our work. Thank you!

Page 2:

1. Authors state x-Cas9 could not edit the zebrafish genome (unpublished data). However, it is not true, I am aware of few zebrafish labs using x-Cas9 on NGT and similar PAM sites, albeit at lower rates. Would you please provide data to support this claim?

Response: Thank you for pointing this inaccurate statement. Actually, we had chosen some sites targeted by SpGCas9 variant with high activities to evaluate the ability of x-Cas9 (purified protein combined with MS-modified gRNA). The data (Figure for reviewers only, Fig.rev1) showed that x-cas9 had the limited ability to edit zebrafish genome.

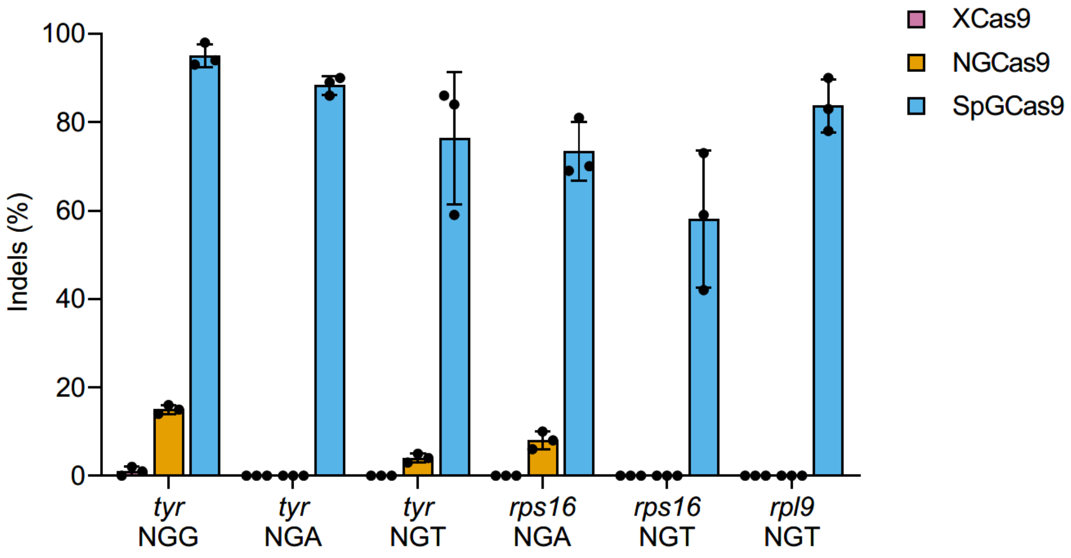

Fig. rev1. Comparison of the editing efficiency of XCas9, NG-Cas9 and SpGCas9 at the same sites targeting NGN PAMs in zebrafish. Editing efficiency was assessed by ICE Tools analysis (n = 3).

2. Cas9-NG has been shown to edit the genome at a higher rate than the x-Cas9 in several species. Did the authors compare the activities of Cas9-NG and SpG?

Response: Per your suggestion, we did these experiments. Cas9-NG indeed exhibited a little higher editing rate than X-cas9 in zebrafish, but still had a limited ability to edit zebrafish genome compared with SpG-Cas9 (Fig.rev1). The purified protein combined with MS-modified gRNA was used in all the above experiments.

3. The data presented on tyr targeting on canonical site is surprising (Figure 1C) given several groups have shown almost 100% efficiency with protein and 80-90% with mRNA in generating albino animals. Therefore, I would have expected a significant improvement between mRNA and protein.

Response: Thank you for pointing out this mistake. After we carefully examined the experiment, we found the Spcas9 proteins used had been stored for a long time. So we expressed and purified the new Spcas9 proteins and did the experiments again. Almost 100% albino embryos could be observed at this time and we updated the Figure1c in the revised manuscript.

4. Add a reference to SpCas9 mRNA and protein comparison.

Response: Thank you. We have cited a reference in the revised manuscript（reference 17, Line 410-411 in our revised manuscript）.

5. Authors show SpG can edit the genome at NGA and NGG PAM with variable efficiencies using

RNP, and it is surprising that no activities were detected using in vitro synthesized guides on non-canonical PAM. This data is also contradicting previously published data in a preprint by the Moreno-Mateos group. This preprint has demonstrated the use of IVT guides at various PAMs as recognized by SpG Cas9 or SpRY. Probably, testing some of those guides will be useful.

Response: We appreciate your suggestion. Actually, SpG could edit zebrafish genome using in vitro synthesized gRNA. Besides the data showed in Figure1d, we provided more data (plus three loci published by Moreno-Mateos group, red frame) to support this (Fig. rev2). Although SpG exhibits activities at these sites using in vitro synthesized gRNA, more sites could only be targeted when using the synthesized MS-modified gRNA but not IVT gRNA. (Fig1f and S12). Our paper provides a more general and efficient method to edit zebrafish genome.

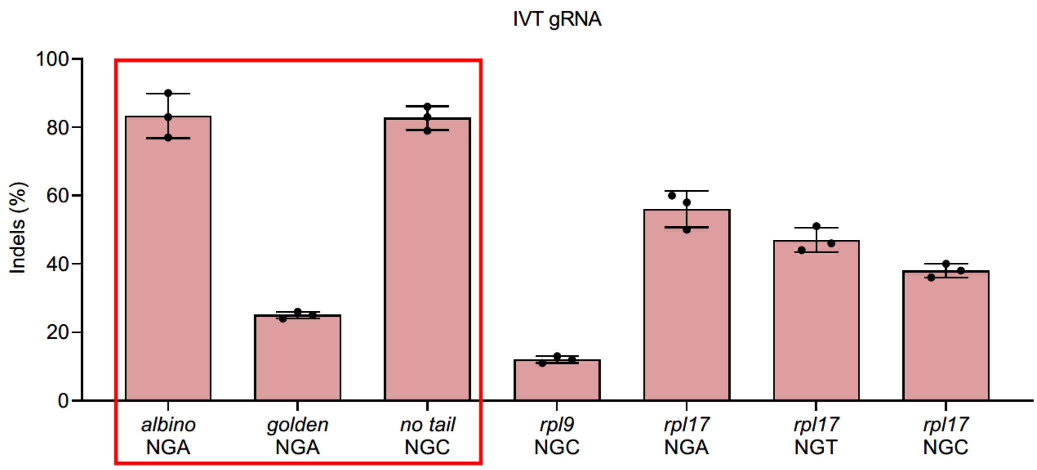

Fig. rev2. SpGCas9 protein with gRNA synthesized *In vitro* showed considerable activities in zebrafish. Three target sites published by Moreno-Mateos group were outlined with a red frame.

6. Mutagenesis activities were measured by Sanger sequencing followed by ICE indel analysis. ICE tool does not recognize the non-canonical PAMs. Therefore, I believe the mutagenesis efficiencies are not accurate. I would suggest either performing next-gen sequencing or clonal sequencing if authors choose to use Sanger sequencing.

Response: We appreciate your valuable comments. Following your suggestions, we performed next-generation sequencing to analyze the indels efficiency. For example, in the ddx21-NGC group, we found the efficiency analyzed by next-generation sequencing (15.35%) was comparable to the result (18.50%) analyzed by ICE tool (Fig. rev3 and Fig.1f), demonstrating that that ICE Analysis Tool can be used to analyze the SpG or SpRY induced indels in zebrafish. To further figure out whether the algorithm design of ICE software is suitable for non-canonical PAMs, we consulted technical support from the Synthego Company. They said" The ICE Analysis Tool assumes that you are using SpCas9 (S. pyogenes) and is optimized for this application. The algorithm uses the input guide sequence to place the predicted cut site 3 bp upstream of the 3' end of the input sequence. If you wish to analyze other nucleases, you would need to enter a guide sequence to position your expected cut site 3 bp from the end of your sequence. This guide sequence will be used by the tool and while the algorithm

does not enforce any particular PAM site it will give you a warning if an NGG was not found." Although SpG and SpRY recognize the non-canonical PAMs, their indels pattern is the same as SpCas9[1, 2]. We think the mutagenesis efficiencies can at least be analyzed by ICE tool technically, and the assumed PAM (TGA) is the same as our practical PAM (Fig.S3). ICE provides a quick, quantitative, and inexpensive assay to analyze the editing outcomes and has been used by many groups[3, 4, 5].

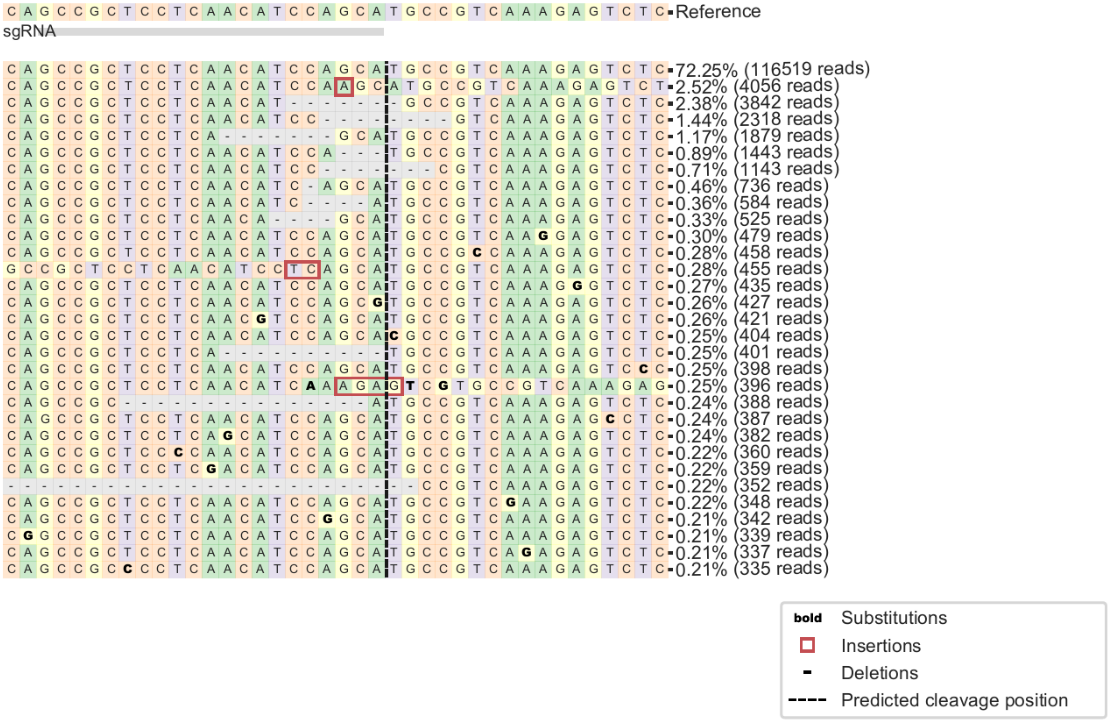

Fig. rev3. On–target analysis of the efficiency of SpG induced cleavage at ddx21-NGC site using next-generation sequencing.

7. Did the authors determine the base editing activities using SpG variant?

Response: It is a good question. We indeed constructed SpG-CBE4max and zSpG-ABE8e and determine the base editing activities in zebrafish. Surprisingly, SpG-CBE4max did not show any activities at the sites (PAM=NGN) that could be targeted by SpRY-CBE4max, the phenomenon was also observed by another group[6]. What's more, the zSpG-ABE8e has the activities to induce A to G conversions, the efficiencies were comparable to zSpRY-ABE8e at the same loci. Considering more relaxed PAM requirements of SpRY variants, we did not show the zSpG-ABE8e data, and recommended zSpRY-ABE8e as a base editing tool in zebrafish.

8. CBE4max is not the most specific base editors, as shown in this manuscript as well. There are many bystander mutations outside of the editing window; therefore, calling it very specific is not supported by the data.

Response: Thanks for your suggestion. We have deleted the sentence in the revised manuscript.

9. How many embryos showed pigment phenotypes at 2dpf following by base editing?

Response: Thanks for your suggestion. We add the number 26/83 in the updated Fig3d.

10. The authors claimed no indels were detected in base editing experiments, but this conclusion is based on Sanger Sequencing of injected embryos. Deep sequencing might shed more light on the specificity of the base editor.

Response: Thank you for your suggestion. To address this comment, we investigated the base editing efficiency of SpRY-CBE4max and zSpRY-ABE8e using deep sequencing. The results showed that SpRY-CBE4max could induce low indels (＜5%) at all three tested locus (Fig. 3e), and lower indels (<0.5%) could be observed at the zSpRY-ABE8e targeted sites (Fig. 4e).

11. Similarly, Sanger sequencing cannot determine whether the ABE8e system can generate high product purity in zebrafish.

Response: Thank you for your suggestion. To address your concern, we analyzed the product purity of zSpRY-ABE8e through NGS ((Fig. 4e and Supplementary Fig. S9-S11). Only A to G base conversion could be observed at all tested sites. Our results indicate that zSpRY-ABE8e system can generate high product purity in zebrafish.

12. In Table S1, around ten targets showed 0% editing. Does this mean either the first base or third base in the PAM influences the editing activities?

Response: It is a nice point. There are ten targets edited by SpRY nuclease that showed 0% editing in Table S1. 8 of 10 sites has a NYN (Y=T or C) PAM sequence. Based on our data, we only observed that SpRY showed a higher preference for NRN than NYN in zebrafish, which is consistent with the results reported in human cells and plants. While, to our knowledge, based on published literatures[2, 7, 8, 9], so far, it is not clear whether the first base or third base in the PAM influences the SpRY nuclease's editing activities or not .As our data is limited, we could not draw any conclusion yet. Additional experiments are needed to more thoroughly characterize the substrate preference of SpRY.

13. Did the authors compare the activities of the base editor using RNP vs mRNA? This experiment is essential given higher efficiencies will allow us to test disease-causing variants rapidly.

Response: We acknowledge your suggestion. We also believe the base editor tools delivered by RNP can improve the efficiency in zebrafish. Actually, we tried to express and purify the SpRY-CBE4max and zSpRY-ABE8e proteins. Unfortunately, the purified proteins did not exhibite any activities in zebrafish. We are still working on it. But anyway, our paper has provided a practical method to edit the targeted sites with up to 96% editing efficiency. In addition, using mRNA will be more convenient in most of labs due to the inconvenience of expressing and purifying proteins.

Minor point:

Please change CRISPR-Cpf1 to CRISPR/Cas12a.

Response: Thank you. We have corrected it in the revised manuscript. (Line 46 in our revised manuscript).

Reviewer #2 (Remarks to the Author):

Notes on: SpG and SpRY variants expand the CRISPR toolbox for genome editing in zebrafish

Remarks to the Author:
In this work, Liang et al. use the recently described SpG and SpRY Cas9 as nucleases and base editor (CBE and ABE) fusions and demonstrate high efficiency on-target editing and high product purity across a wide variety of different PAM sequences thereby demonstrating the usability of these novel PAM-flexible Cas9 variants in zebrafish. Using these tools, the authors were able to generate previously inaccessible genetic variants. Even though the presented results are important, especially the data on SpRY base editing (Figure 3 and 4) should be much more comprehensive both regarding the number of investigated target sites and the presentation of the data. Additionally, important aspects that come to mind when using PAM-flexible Cas9 variants such as the putatively increased risk for off-target editing have not been investigated by the authors. Furthermore, the use of NGS and a more thorough comparison to existing technology would be necessary to make this study of interest to a broader readership.

Response: We truly thank the Reviewer for this positive evaluation on the submitted manuscript, and the constructive suggestions listed below to improve our manuscript. We have revised the manuscript accordingly.

Major comments:

1) Fig 2) It appears that only one spacer is used to assess the impact of a specific PAM sequence. The spacer sequence could be a big confounder on the efficiencies reported in Fig. 2d. The authors should perform this experiment with more spacers e.g. three per particular PAM sequence to be able to draw conclusion on the influence of a particular PAM or alternatively only present the data in collapsed form (Fig. 2e).

Response: Thanks for your suggestion. The spacer sequence is indeed a big confounder on the efficiencies of SpRY nucleases activity in vivo. Actually, we had shown the efficiencies of 32 endogenous genomic sites from two genes (rpl9 and rpl17) which contained all 16 possible alternative PAM sequences in the original manuscript. In order to draw the conclusion more reliable (three per particular PAM sequence), we added other 16 spacers from rps16 locus in the new updated fig.2b and fig.2c.

2) Fig 3) and 4) Instead of showing representative sanger traces, the authors should show barplots analogous to Fig 1) and Fig2) that allow the reader to assess the variance of gene editing.

Response: Thanks for your suggestion. We have updated the Fig.3 and Fig.4 in the revised

manuscript.

3) The authors should also elaborate on why they have chosen a different delivery modality for the base editors as compared to nucleases.

Response: We acknowledge your suggestion. We also believe the base editor tools delivered by RNP can improve the efficiency in zebrafish. Actually, we tried to express and purify the SpRY-CBE4max and zSpRY-ABE8e proteins. Unfortunately, the purified proteins did not exhibit any activities in zebrafish. We are still working on it. But anyway, our paper has provided a practical method to edit the targeted sites with up to 96% editing efficiency. In addition, using mRNA will be more convenient in most of labs due to the inconvenience of expressing and purifying proteins.

4) The authors should include more target sites with a greater diversity of PAM sequences in their assessment of SpRY CBE and ABE gene editing efficiencies.

Response: Thank you for your suggestion. We have added more target sites to assess the editing efficiency of SpRY-CBE4max and zSpRY-ABE8e (Fig.3b and Fig.4b). It is worth noting that SpRY-CBE4max comprising rat APOBEC1 has some limitations, which preferentially targets the cytidines preceded by T (namely the cytidines in the TC motif, the target C underlined), so we did not have more sites to evaluate the base conversion efficiency.

5) The authors should elaborate on the implications of their results in the context of the recently described uses of prime editing in zebrafish.

Response: We acknowledge your suggestion. We had added this part in the discussion in the revised manuscript. (Line 247-250 in our revised manuscript)

6) An analysis of potential off-target effects of SpRY nuclease and base editors is missing. It would be great if the authors could analyse the degree to which off-targets occur with the analysed PAM-flexible Cas9 variants since the increased PAM-flexibility might also increase the risk for off-target gene editing.

Response: Thank you for your suggestion. To address this, we have analyzed the off-target effects of SpG, SpRY nuclease and SpRY-based base editors in zebrafish using deep sequencing (Fi.2d, Fig.3e and Fig.4e). The results showed that only low off-target editing (＜0.5%) could be detected in all the above situation. The details can be found in the revised manuscript. (Line 102-106, 121-123, 157-158, 186-187 in our revised manuscript)

7) It would be great if the authors could compare SpRY to other PAM-flexible variants that have been recently described (e.g. Nishimasu et al., Science, 2018).

Response: Thank you for your comments. Following your suggestions. We chose some sites edited by SpG-Cas9 with high efficiency to assess the X-Cas9 and Cas9-NG activities in zebrafish. The results

showed that both X-Cas9 and Cas9-NG exhibited a limited ability to edit zebrafish genome compared with SpG-Cas9 (Figure for reviewers only, Fig.rev1). The purified protein combined with MS-modified gRNA was used in all the above experiments.

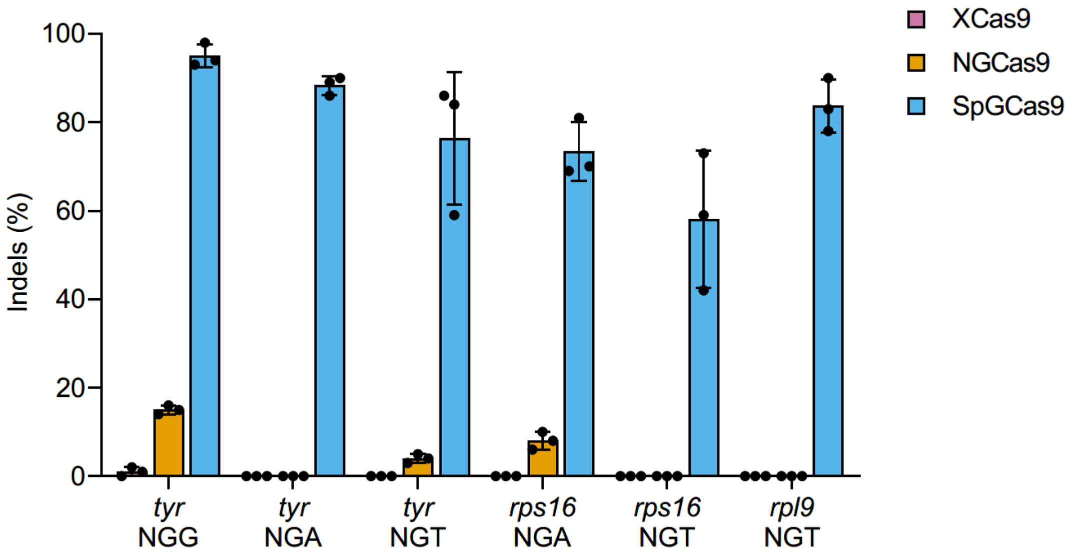

Fig. rev1. Comparison of the editing efficiency of XCas9, NG-Cas9 and SpGCas9 at the same sites targeting NGN PAMs in zebrafish. Editing efficiency was assessed by ICE Tools analysis (n = 3).

8) It would be advisable to perform NGS on at least some of the base edited samples to comprehensively assess the purity of base editing products.

Response: Thank you for your suggestion. To address your concern, we analyzed the product purity of SpRY-CBE4max and zSpRY-ABE8e through NGS. In the SpRY-CBE4max system, the targeted cytidines were mainly converted to thymine, and low conversion rate of adenine and guanine could also be detected (Fig. 3e and Supplementary Fig. S5-S7). But in zSpRY-ABE8e system, only A to G base conversion could be observed at all tested sites (Fig. 4e and Supplementary Fig. S9-S11).

9) It could be helpful if the authors would compare SpRY CBE and ABE base editing to SpRY-mediated HDR in terms of editing efficiency and product purity. The authors should then give recommendations on which technology to use in which cases.

Response: Thanks for your suggestion. Although the HDR-based modification has been widely used in human cells and mammals, but the efficiency is still very poor in zebrafish. To make it more clearly for readers, we try to compare the efficiency between HDR and base editor at the same locus. Most publications report on the use of short (50–136 nt), single stranded oligonucleotide templates for HDR in zebrafish[10, 11, 12]. Here, using the same gRNA (mitfa-E255K-NAT), we injected a 120bp ssDNA donor template and SpRY nuclease into zebrafish embryos, and analyzed the efficiency of HDR-based modification through NGS. As expected, the efficiency is quiet low (0.68%) and large

amounts of indels (15.08%) could be detected, which is not comparable to SpRY-CBE4max induced base conversion (C5T, 11.72%, indels (1.69%)) at this locus (Fig. rev4, Fig.3e and Supplementary Fig. S7). We cannot simply say the base editor system is better than HDR, due to the complexity of HDR. In addition, the success rate for modifying the genome in zebrafish is inconsistent due to a variety of protocols. What's more, many factors such as type of template, length of the homology arms, symmetry of repair template, choice of endonuclease, endonuclease mRNA or protein and targeted strand affects the HDR efficiency[13]. Optimizing the efficiency of HDR is a very complex thing, which is beyond the scope of this paper. As recommendations for constructing the precise disease model in zebrafish, if one case can be targeted by CBE or ABE base editors, the first choice is them, due to their high efficiency, high product purity and convenience; if the intended base conversion cannot be accessible by base editors, you can choose the prime editing or HDR. We had added this part in the discussion part in the revised manuscript. (Line 227-250 in our revised manuscript).

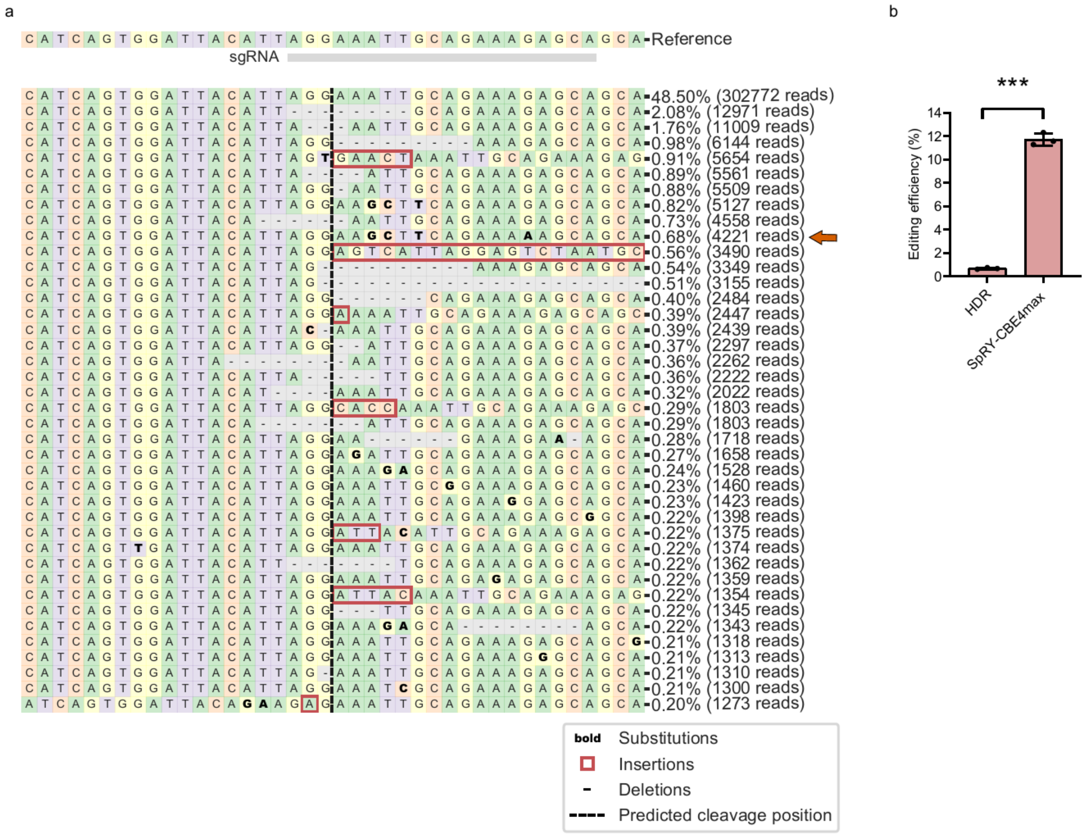

Fig. S13. NGS analysis of precise point mutation introduction to *mitfa*. a. Analysis of HDR-induced C to T editing at mitfa. Red arrow indicated the correct HDR reads. b. Bar plot comparation of HDR- or SpRY-CBE4max-induced C to T editing at *mitfa* sites.

Minor comments:

1) Individual data points should be shown for all figures.

Response: Thank you for your suggestion. We had updated all figures in the revised manuscript.

2) Fig 1d. is not a histogram as stated in the figure legend.

Response: Thank you for your suggestion. We had corrected it in the revised manuscript.

Reference

1. Jeremy Vicencio CS-B, Ismael Moreno-Sánchez, David Brena, Dmytro Kukhtar, Miguel Ruiz-López, Mariona Cots-Ponjoan, Charles E. Vejnar, Alejandro Rubio, Natalia Rodrigo Melero, Carlo Carolis, Antonio J. Pérez-Pulido, Antonio J. Giráldez, Benjamin P. Kleinstiver, Julián Cerón,. Genome editing in animals with minimal PAM CRISPR-Cas9 enzymes. *bioRxiv* 2021.

2. Walton RT, Christie KA, Whittaker MN, Kleinstiver BP. Unconstrained genome targeting with near-PAMless engineered CRISPR-Cas9 variants. *Science* 2020, **368**(6488)**:** 290-296.

3. Eleveld TF, Bakali C, Eijk PP, Stathi P, Vriend LE, Poddighe PJ*, et al.* Engineering large-scale chromosomal deletions by CRISPR-Cas9. *Nucleic acids research* 2021, **49**(21)**:** 12007-12016.

4. Wen W, Quan ZJ, Li SA, Yang ZX, Fu YW, Zhang F*, et al.* Effective control of large deletions after double-strand breaks by homology-directed repair and dsODN insertion. *Genome biology* 2021, **22**(1)**:** 236.

5. Zhao D, Jones JL, Gasperini RJ, Charlesworth JC, Liu GS, Burdon KP. Rapid and efficient cataract gene evaluation in F0 zebrafish using CRISPR-Cas9 ribonucleoprotein complexes. *Methods* 2021.

6. Marion Rosello MS, Marina C Mione, Jean-Paul Concordet, Filippo Del Bene. Disease modeling by efficient genome editing using a near PAM-less base editor in vivo. *bioRxiv* 2021.

7. Xu Z, Kuang Y, Ren B, Yan D, Yan F, Spetz C*, et al.* SpRY greatly expands the genome editing scope in rice with highly flexible PAM recognition. *Genome biology* 2021, **22**(1)**:** 6.

8. Ren Q, Sretenovic S, Liu S, Tang X, Huang L, He Y*, et al.* PAM-less plant genome editing using a CRISPR-SpRY toolbox. *Nature plants* 2021, **7**(1)**:** 25-33.

9. Li J, Xu R, Qin R, Liu X, Kong F, Wei P. Genome editing mediated by SpCas9 variants with broad non-canonical PAM compatibility in plants. *Molecular plant* 2021, **14**(2)**:** 352-360.

10. Prykhozhij SV, Fuller C, Steele SL, Veinotte CJ, Razaghi B, Robitaille JM*, et al.* Optimized knock-in of point mutations in zebrafish using CRISPR/Cas9. *Nucleic acids research* 2018, **46**(17)**:** 9252.

11. Tessadori F, Roessler HI, Savelberg SMC, Chocron S, Kamel SM, Duran KJ*, et al.* Effective CRISPR/Cas9-based nucleotide editing in zebrafish to model human genetic cardiovascular disorders. *Disease models & mechanisms* 2018, **11**(10).

12. Armstrong GA, Liao M, You Z, Lissouba A, Chen BE, Drapeau P. Homology Directed Knockin of

Point Mutations in the Zebrafish tardbp and fus Genes in ALS Using the CRISPR/Cas9 System. *PloS one* 2016, **11**(3)**:** e0150188.

13. Prill K, Dawson JF. Homology-Directed Repair in Zebrafish: Witchcraft and Wizardry? *Frontiers in molecular biosciences* 2020, **7:** 595474.

Reviewers' Comments:

Reviewer #1:
Remarks to the Author:
The authors did a fantastic job in addressing most of my points, and these revisions have significantly improved the manuscript. I only have a few minor points that would help the community to utilize their methods widely, and bring them citations.

1) I am not sure why did authors did not choose other loci. In my opinion, adding 3-4 more loci and using the most successful PAM will give confidence to the users to use their method. You do not need to do next-generation sequencing, Sanger sequencing should be enough.

2) Looks like authors have generated SpG-base editors, I would recommend using more sites with highly successful PAM sequences from their dataset. Since you already generated data for a few loci, please include it here.

3)NAA PAM could be useful in targeting non-coding regions, showing a couple of targets will be fantastic.

4) Authors stated, "SpRY-CBE4max comprising rat APOBEC1 has some limitations, which preferentially targets the cytidines preceded by T (namely the cytidines in the TC motif, the target C underlined), so we did nothave more sites to evaluate the base conversion efficiency." what do they mean by this? They could have chosen other sites in a different gene.

Overall good work!

Reviewer #2:
Remarks to the Author:
The authors have addressed several comments satisfactorily, however multiple aspects of the manuscript could benefit from a more careful investigation and additional data.

Major comments replies:

1) The authors have addressed this by adding an additional replicate.

2) The authors have addressed this by presenting the data as a barplot.

3) The SpRY delivery modality (mRNA vs. RNP) could influence the targetability of PAM sequences due to prolonged cellular persistence of mRNA-delivered SpRY. If SpRY base editors cannot be purified, the authors should compare the efficiencies of mRNA-delivered SpRY nuclease vs. RNP-delivered SpRY nuclease at multiple target sites with various PAM sequences.

4) Analogous to the nuclease data, every PAM sequence (16 total) should be assessed using two – three spacers for CBE and ABE.

5) Sufficiently addressed.

6) The authors should explain how the “three most likely off-target sites” were predicted by cas-offinder. To my knowledge cas-offinder does not provide an off-target ranking. A conservative off-target analysis approach usually includes investigation of all genomic sequences up to (and including) three mismatches to the on-target site. It would make the off-target analysis more comprehensive if the authors could do this for some gRNAs. In addition, it would be helpful if the authors could specify the number of genomic sites with up to (and including) three mismatches next to the deep-sequencing off-target data for the different gRNAs to put the number of deep-sequenced genomic sites into context.

So far, given that a low number of genomic sites was investigated and that for rpl17-NTA, two of

three genomic sites showed off-target editing, it is questionable if the authors can conclude that off-targets were “negligible” and that zSpRY-ABE8e is “highly specific”.

7) Sufficiently addressed. Should be included in manuscript.

8) Sufficiently addressed.

Additional Minor comments:

1) Alignment of bars at bottom of Fig. 3b is off.

2) Fig. 3 b says SpRY-CBE4max nuclease. Correct would be SpRY-CBE4max nickase or SpRY-CBE4max editor.

3) Grammar throughout the manuscript could be improved.

Response to Reviewers

We thank the reviewers and editor for constructive critiques. We revised the manuscript accordingly. The following specific answers to each question should also address concerns summarized by the editors. We think the paper is improved and hope it is acceptable for publication in Nature Communications. A copy of track changes is also included.

REVIEWER COMMENTS

Reviewer #1 (Remarks to the Author):

The authors did a fantastic job in addressing most of my points, and these revisions have significantly improved the manuscript. I only have a few minor points that would help the community to utilize their methods widely, and bring them citations.

Response: We truly thank you for the positive feedback and highly appreciate the recognition of the significance of our work. Thank you!

1. I am not sure why did authors did not choose other loci. In my opinion, adding 3-4 more loci and using the most successful PAM will give confidence to the users to use their method. You do not need to do next-generation sequencing, Sanger sequencing should be enough.

Response: Thanks for your suggestion. We have changed tyr locus to rpl17 locus which showed higher activity in updated Fig.1d. We think this will give confidence to the users to use our method.

2. Looks like authors have generated SpG-base editors, I would recommend using more sites with highly successful PAM sequences from their dataset. Since you already generated data for a few loci, please include it here.

Response: Per your suggestion, we have added this data (Fig. S18) in our revised manuscript.

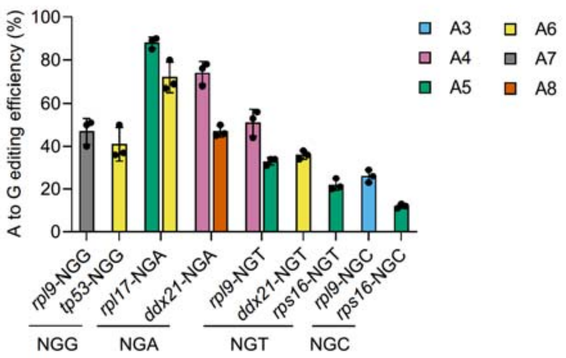

Fig. S18. Summary of A-to-G base editing efficiency of various loci with NGN PAMs induced by zSpG-ABE8e editor in zebrafish. The position of editing base in the gRNA was labelled with numbers.

3. NAA PAM could be useful in targeting non-coding regions, showing a couple of targets will be fantastic.

Response: It is a nice point. We added 2 and 3 more targets with the NAA PAM in SpRY-CBE4max (updated Fig.3b) and zSpRY-ABE8e group (updated Fig.4b) respectively.

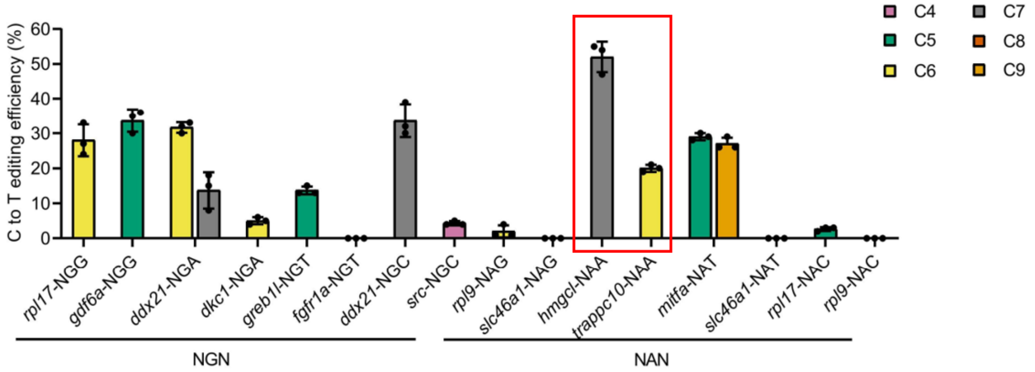

Fig. 3b. Summary of C-to-T base editing efficiency of various loci with NRN PAMs induced by SpRY-CBE4max editor in zebrafish. The position of the editing base in the gRNA was labelled with numbers. Two target sites with NAA PAM were outlined with a red frame.

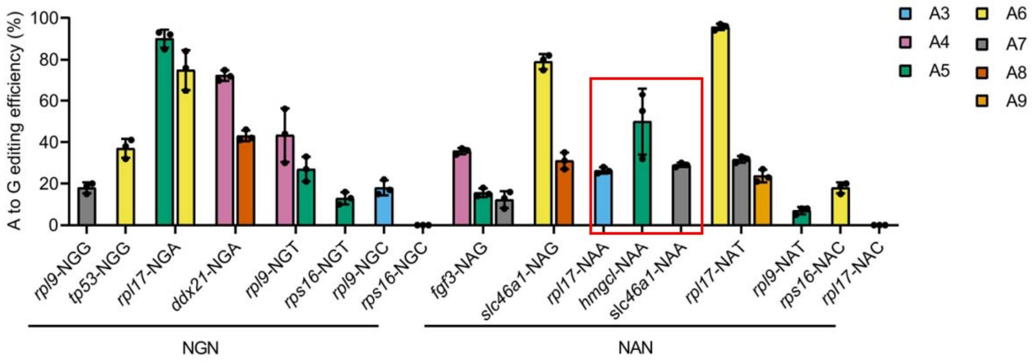

Fig. 4b. Summary of A-to-G base editing efficiency of various loci with NRN PAMs induced by zSpRY-ABE8e editor in zebrafish. The position of editing base in the gRNA was labelled with numbers. Three target sites with NAA PAM were outlined with a red frame.

4. Authors stated, "SpRY-CBE4max comprising rat APOBEC1 has some limitations, which preferentially targets the cytidines preceded by T (namely the cytidines in the TC motif, the target C underlined), so we did not have more sites to evaluate the base conversion efficiency." what do they mean by this? They could have chosen other sites in a different gene.

Response: Thank you for pointing this inaccurate statement "so we did not have more sites to evaluate the base conversion efficiency". We have chosen more sites from different genes to assess the base conversion efficiency of SpRY-CBE4max (updated Fig.3b and Fig.S6).

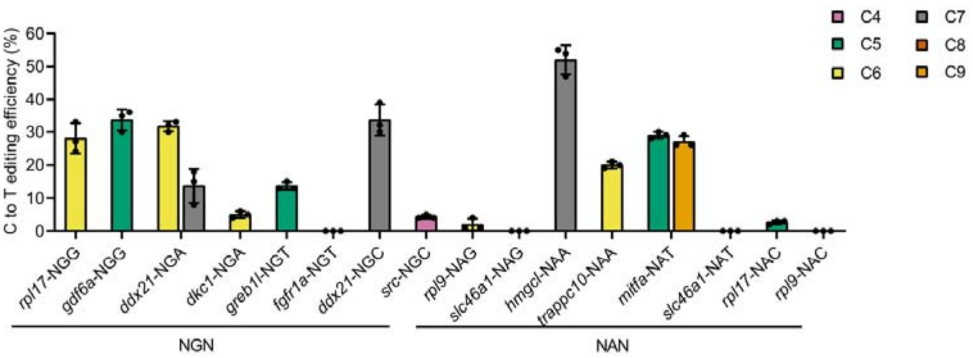

Fig. 3b. Summary of C-to-T base editing efficiency of various loci with NRN PAMs induced by SpRY-CBE4max editor in zebrafish. The position of the editing base in the gRNA was labelled with numbers.

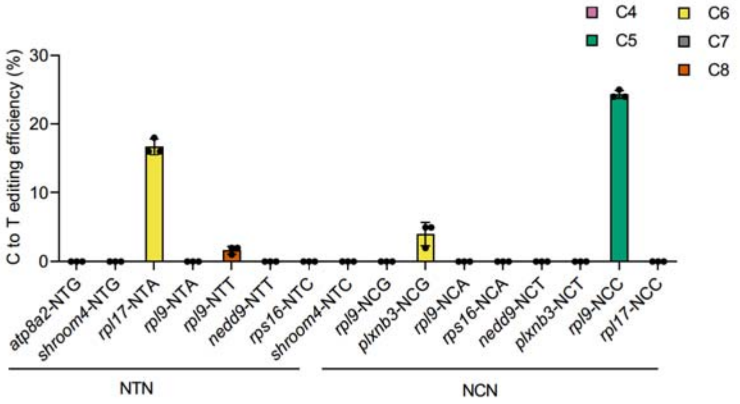

Fig. S6. Summary of C-to-T base editing efficiency of various loci with NYN PAMs induced by SpRY-CBE4max editor in zebrafish. The position of the editing base in the gRNA was labelled with numbers.

Reviewer #2 (Remarks to the Author):

The authors have addressed several comments satisfactorily, however multiple aspects of the manuscript could benefit from a more careful investigation and additional data.

Response: We truly thank you for this positive evaluation on the resubmitted manuscript, and the constructive suggestions listed below to improve our manuscript. We have revised the manuscript accordingly.

Major comments replies:

1) The authors have addressed this by adding an additional replicate.

Response: Thank you!

2) The authors have addressed this by presenting the data as a barplot.

Response: Thank you!

3) The SpRY delivery modality (mRNA vs. RNP) could influence the targetability of PAM sequences due to prolonged cellular persistence of mRNA-delivered SpRY. If SpRY base editors cannot be purified, the authors should compare the efficiencies of mRNA-delivered SpRY nuclease vs. RNP-delivered SpRY nuclease at multiple target sites with various PAM sequences.、

Response: We acknowledge your suggestion. We compared the editing efficiency of mRNA-delivered SpRY nuclease vs. RNP-delivered SpRY nuclease at multiple target sites with various PAM sequences. The results showed that SpRY protein had higher or similar activity compared with mRNA-delivered SpRY nuclease in zebrafish (Fig.S15).

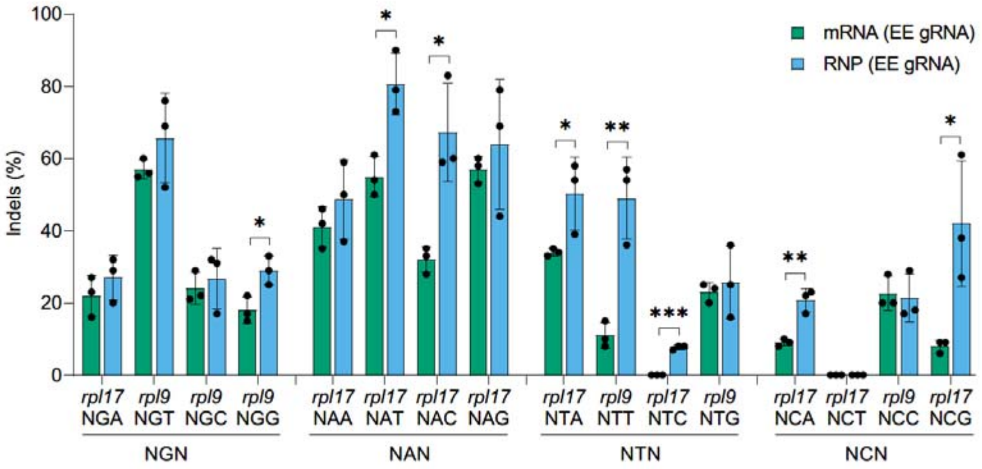

Fig. S15. Comparison of the editing efficiency of SpRY protein and mRNA with EE gRNA at the same sites targeting NNN PAMs in zebrafish. Editing efficiency was assessed by ICE Tools analysis (n = 3).

4) Analogous to the nuclease data, every PAM sequence (16 total) should be assessed using two – three spacers for CBE and ABE.

Response: Thank you for your suggestion. We have added at least two spacers with different PAM sequence to assess the activity of CBE and ABE (updated Fig.3b, Fig.S6, Fig4b and Fig.S10).

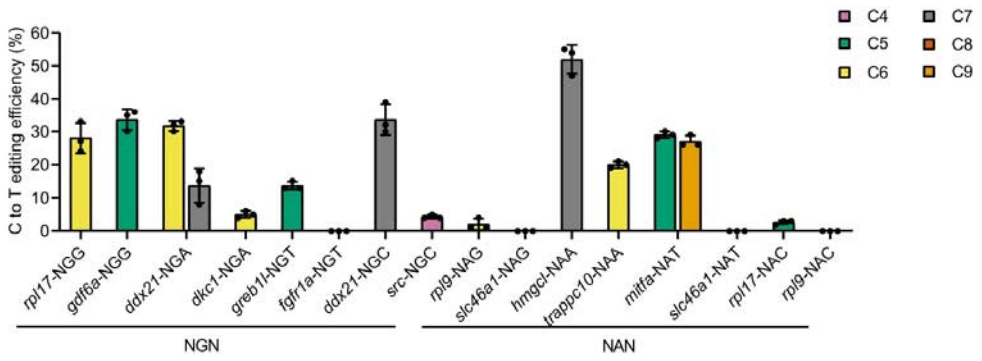

Fig. 3b. Summary of C-to-T base editing efficiency of various loci with NRN PAMs induced by SpRY-CBE4max editor in zebrafish. The position of the editing base in the gRNA was labelled with numbers.

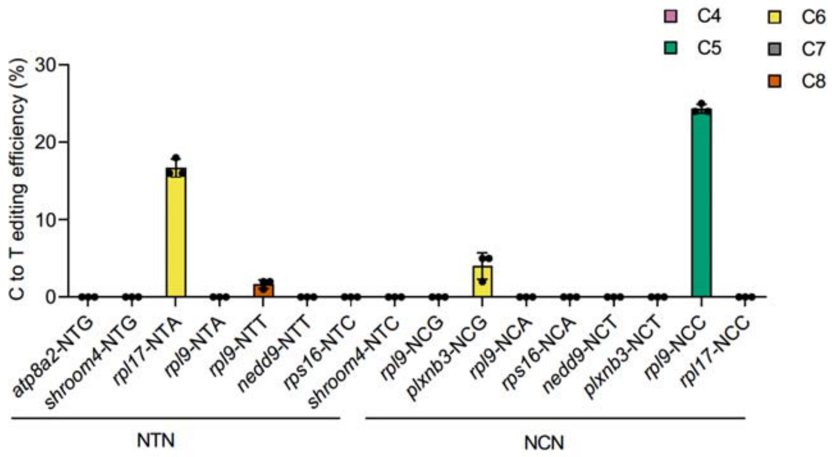

Fig. S6. Summary of C-to-T base editing efficiency of various loci with NYN PAMs induced by SpRY-CBE4max editor in zebrafish. The position of the editing base in the gRNA was labelled with numbers.

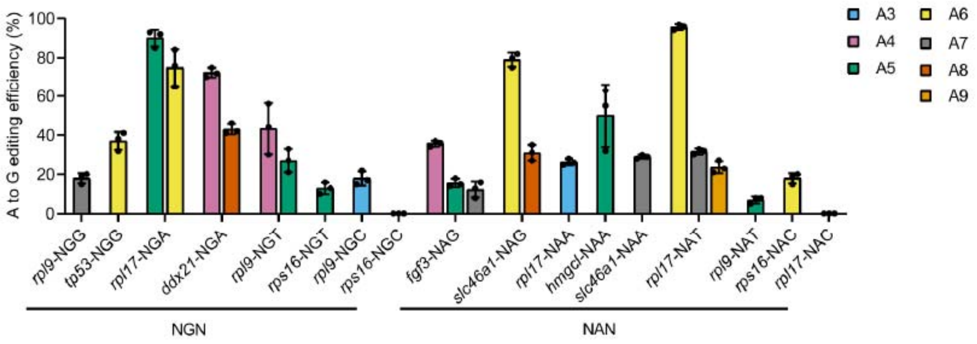

Fig. 4b. Summary of A-to-G base editing efficiency of various loci with NRN PAMs induced by zSpRY-ABE8e editor in zebrafish. The position of editing base in the gRNA was labelled with numbers.

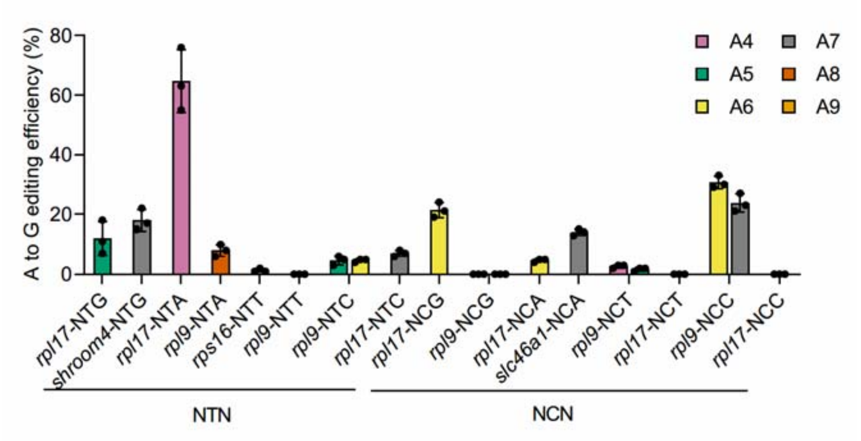

Fig. S10. Summary of A-to-G base editing efficiency of various loci with NYN PAMs induced by zSpRY-ABE8e editor in zebrafish. The position of the editing base in the gRNA was labelled with numbers.

5) Sufficiently addressed.

Response: Thank you!

6) The authors should explain how the "three most likely off-target sites" were predicted by cas-offinder. To my knowledge cas-offinder does not provide an off-target ranking. A conservative off-target analysis approach usually includes investigation of all genomic sequences up to (and including) three mismatches to the on-target site. It would make the off-target analysis more comprehensive if the authors could do this for some gRNAs. In addition, it would be helpful if the authors could specify the number of genomic sites with up to (and including) three mismatches next to the deep-sequencing off-target data for the different gRNAs to put the number of deep-sequenced genomic sites into context.

So far, given that a low number of genomic sites was investigated and that for rpl17-NTA, two of three genomic sites showed off-target editing, it is questionable if the authors can conclude that off-targets were "negligible" and that zSpRY-ABE8e is "highly specific".

Response: Thank you for pointing this inaccurate statement. The off-target sites for each gRNA were predicted by Cas9-OFFinder[1] and CRISPOR[2]. The specificity score was calculated by CRISPOR[2] and then we chose three most likely off-target sites (the top 3 high-scoring sites) to assess the off-target effect. Usually, there are dozens of off-target sites for each gRNA, considering the cost, many groups including us chose limited sites to assess the off-target effect[3, 4, 5, 6, 7, 8]. According to your suggestions, to make it more clearly for readers, we provided all the predicted off-target sites for gRNAs we chose (Supplementary Table S8) and the number of reads of NGS in each group (updated Fig.2d, Fig.3e, Fig.4e and Supplementary Table S2). What's more, for preciseness, we deleted the sentence "The negligible off-target effects further demonstrated zSpRY-ABE8e system was a very specific system" in the revised manuscript.

gRNA	Off-target site	Mismatched bases (bp)	Genome location	Sequence	Efficiency (reads)
rpl17-NTA	1	2	14: 2558561	CACATCCGCAtGGCTgACAAAAG	0% (0/112737)
	2	3	14: 15538790	gAtATCtGCAAGGCTAACAATAT	0% (0/163177)
	3	3	4: 8994566	CcCATtaGCAAGGCTAACAAGGT	0% (0/76022)
rpl9-NCC	1	3	20: 35366307	CtaaCAAGGGCCGCACAGTTGCA	0% (0/117133)
	2	3	20: 45550601	tCCTggAGGGCCGCACAGTTTAG	0% (0/103267)
	3	3	19: 31596485	CgCTCcAGtGCCGCACAGTTCTG	0% (0/124259)
rpl17-NAT	1	3	7: 45367366	AaaCTAgCAAGTACCTGAAGTCA	0% (0/102273)
	2	3	15: 4665921	AGaCgAgCAAGTACCTGAAGAGC	0% (0/138421)
	3	3	9: 22868125	AcGCTAttAAGTACCTGAAGGCT	0% (0/99936)

Fig. 2d. Detection of mutation at potential off-target sites induced by SpRYCas9 nuclease at three loci using NGS.

gRNA	Target site	Mismatched bases (bp)	Genome location	Sequence	Efficiency (reads)	Indels (reads)
rpl17-NTA	on target	0	21: 19026624	CACATCCGCAAGGCTAACAAGTA	C6T, 17.60% (26111/148322) C7T, 1.30% (1930/148322)<br>C6A, 1.07% (1580/148322) C7A, 0% (0/148322)<br>C6G, 0.27% (349/148322) C7G, 0% (0/148322)	0.28% (420/148322)
	off target-1	2	14: 2558561	CACATCCGCAtGGCTgACAAAAG	0% (0/69074)	NA
	off target-2	3	14: 15538790	gAtATCtGCAAGGCTAACAATAT	C6T, 0.27% (324/118829)	NA
	off target-3	3	4: 8994566	CcCATtaGCAAGGCTAACAAGGT	0% (0/76526)	NA
ddx21-NGA	on target	0	13: 199689	AACATCCAGCATGCCGTCAAAGA	C6T, 29.58% (43604/147392) C7T, 20.61% (30371/147392)<br>C6A, 3.38% (4982/147392) C7A, 0.60% (888/147392)<br>C6G, 2.65% (3900/147392) C7G, 0% (0/147392)	4.61% (6794/147392)
	off target-1	3	1: 23158926	AgCAcaCAGCATGCCGTCAACGT	0% (0/111961)	NA
	off target-2	3	20: 4853100	AACATCaAGCATGagGTCAAAGC	0% (0/126102)	NA
	off target-3	3	9: 26319599	AAaAcCCAGCATGCaGTCAAAGA	0% (0/105120)	NA
mitfa-NAT	on target	0	6: 43434623	TGCTCTTTCTGCAATTTCCTAAT	C5T, 11.72% (27693/150885) C9T, 10.81% (16316/150885)<br>C5A, 0.89% (1284/150885) C9A, 0.98% (1472/150885)<br>C5G, 0.45% (686/150885) C9G, 0.25% (381/150885)	1.69% (2565/150885)
	off target-1	1	20: 40305404	TGCTtTTTCTGCAATTTCCTCTC	0% (0/115601)	NA
	off target-2	1	11: 23204423	TGCTCTTTCTGCAATaTCCTGTT	0% (0/121029)	NA
	off target-3	2	5: 67425544	TGtTCcTTCTGCAATTTCCTTTG	0%(0/121044)	NA

Fig. 3e. On-target, product purity and Off-target analysis of SpRY-CBE4max induced C-to-T editing at *rpl17*-NTA, *ddx21*-NGA and *mitfa*-NAT sites using NGS.

gRNA	Target site	Mismatched bases (bp)	Genome location	Sequence	Efficiency	Indels
rpl17-NTA	on target	0	21: 19026624	CACATCCGCAAGGCTAACAAGTA	A4G, 54.66% (148528/270275)	0% (0/270275)
	off target-1	2	14: 2558561	CACATCCGCAtGGCTgACAAAAG	0% (0/167451)	NA
	off target-2	3	14: 15538790	gAtATCtGCAAGGCTAACAATAT	A2G, 0.24% (518/214225)	NA
	off target-3	3	4: 8994566	CcCATtaGCAAGGCTAACAAGGT	A4G, 0.21% (370/176358)	NA
rpl9-NGT	on target	0	1: 22583134	CTCAAGGGCCGCACAGTTACCGT	A4G, 58.95% (632742/1073631)<br>A5G, 32.91% (353250/1073631)<br>A13G, 0.46% (4935/1073631)	0%(0/1073631)
	off target-1	3	21: 14193066	CTCAAtGGCaGCACAGTgACAGT	0% (0/191477)	NA
	off target-2	3	11: 5459995	CTCAAGGGCCaCACAGTagCAGG	0% (0/188479)	NA
	off target-3	3	23: 32000757	CTCAAGGaCtGCAgAGTTACAGC	0% (0/223643)	NA
tsr2-NGT	on target	0	8: 8307419	CAAGATGGCGCCGCTCTCATCGT	A5G, 56.93% (175646/306796)	0.45% (1380/306796)
	off target-1	3	22: 15831496	CAAaATaGCGgCGCTCTCATTGG	0% (0/233878)	NA
	off target-2	3	1: 42311156	CAAGATGGCaCCttTCTCATTGT	0% (0/313409)	NA
	off target-3	3	18: 32122506	CAAGATGGCGCtGCTCTgcTGGC	0% (0/247819)	NA

Fig. 4e. On-target, product purity and Off-target analysis of zSpRY-ABE8e induced A-to-G editing at *rpl17*-NTA, *rpl9*-NGT and *tsr2*-NGT sites using NGS.

Table S2. Off-target analysis of SpG nuclease.

gRNA	Off-target site	Mismatched bases(bp)	Genome location	Sequence (N20+PAM)	Efficiency (reads)
rpl17-NGA	1	3	8:30668379	TAAtAAGaAaCTGAAGGATGAGG	0.50% (707/141401)
	2	3	22:3883532	TAAtAAtTAtCTGAAGGATGAGC	0.48% (532/110817)
	3	3	20:20939945	aAACAtGTACaTGAAGGATGTGT	0% (0/142580)
rpl9-NGT	1	3	21:14193066	CTCAAtGGCaGCACAGTgACAGT	0% (0/136601)
	2	3	11:5459995	CTCAAGGGCCaCACAGTagCAGG	0% (0/118727)
	3	3	23:32000757	CTCAAGGaCtGCAgAGTTACAGC	0% (0/140651)
ddx21-NGC	1	2	20:29906243	CCGCTCtTCgACATCCAGCATGG	0% (0/152282)
	2	3	10:10992759	CCtCTCCTCttCATCCAGCATGT	0% (0/123749)
	3	2	11:30494286	CCcCTCCTCAACATCCAGaATGT	0% (0/132346)

7) Sufficiently addressed. Should be included in manuscript.

Response: Thank you! We have added this data in the revised manuscript.

8) Sufficiently addressed.

Response: Thank you!

Additional Minor comments:

1) Alignment of bars at bottom of Fig. 3b is off.

Response: It is done. We have updated the Fig.3b.

2) Fig. 3 b says SpRY-CBE4max nuclease. Correct would be SpRY-CBE4max nickase or SpRY-CBE4max editor.

Response: Thank you for your suggestion. We have corrected it.

3) Grammar throughout the manuscript could be improved.
Response: Thank you! We have revised the grammar throughout the manuscript.

References

1. Bae S, Park J, Kim JS. Cas-OFFinder: a fast and versatile algorithm that searches for potential off-target sites of Cas9 RNA-guided endonucleases. *Bioinformatics* 2014, **30**(10)**:** 1473-1475.

2. Concordet JP, Haeussler M. CRISPOR: intuitive guide selection for CRISPR/Cas9 genome editing experiments and screens. *Nucleic acids research* 2018, **46**(W1)**:** W242-W245.

3. Ren Q, Sretenovic S, Liu S, Tang X, Huang L, He Y*, et al.* PAM-less plant genome editing using a CRISPR-SpRY toolbox. *Nature plants* 2021, **7**(1)**:** 25-33.

4. Matson AW, Hosny N, Swanson ZA, Hering BJ, Burlak C. Optimizing sgRNA length to improve target specificity and efficiency for the GGTA1 gene using the CRISPR/Cas9 gene editing system. *PloS one* 2019, **14**(12)**:** e0226107.

5. Singh K, Evens H, Nair N, Rincon MY, Sarcar S, Samara-Kuko E*, et al.* Efficient In Vivo Liver-Directed Gene Editing Using CRISPR/Cas9. *Molecular therapy : the journal of the American Society of Gene Therapy* 2018, **26**(5)**:** 1241-1254.

6. Yu S, Yao Y, Xiao H, Li J, Liu Q, Yang Y*, et al.* Simultaneous Knockout of CXCR4 and CCR5 Genes in CD4+ T Cells via CRISPR/Cas9 Confers Resistance to Both X4- and R5-Tropic Human

Immunodeficiency Virus Type 1 Infection. *Human gene therapy* 2018, **29**(1)**:** 51-67.

7. Hay EA, Khalaf AR, Marini P, Brown A, Heath K, Sheppard D*, et al.* An analysis of possible off target effects following CAS9/CRISPR targeted deletions of neuropeptide gene enhancers from the mouse genome. *Neuropeptides* 2017, **64:** 101-107.

8. Qin W, Lu X, Liu Y, Bai H, Li S, Lin S. Precise A*T to G*C base editing in the zebrafish genome. *BMC biology* 2018, **16**(1)**:** 139.
